# Supplementary material for: Characterization of alternative mRNA splicing in cultured cell populations representing progressive stages of human fetal kidney development
Source: Sci Rep. 2022 Nov 15;12:19548. doi: 10.1038/s41598-022-24147-z (PMC9666651; doi:10.1038/s41598-022-24147-z)
Supplement: Supplementary file 1 — Supplementary Legends. [file 41598_2022_24147_MOESM1_ESM.docx]

**APPENDICES**

**Supplementary information:** Supplementary text and figures.

**Table S1:** Gene expression values (raw and normalized counts).

**Table S2:** Gene Ontology (GO) enrichment analysis results from ToppGene for the set of 395 genes that were found by intersecting all the genes that were upregulated at least 2-fold (log2foldChange > 1) in hFK3 (the mature fetal developmental fraction) with respect to hFK1 (the immature fraction), WT11, WT14, and WT37 (see Figs. 2D and S1).

**Table S3:** rMATS tables of cassette/skipped exons (SE).

**Table S4:** rMATS tables of mutually exclusive exons (MXE).

**Table S5:** rMATS tables of alternative 3’ splice-sites (A3SS).

**Table S6:** rMATS tables of alternative 5’ splice-sites (A5SS).

**Table S7:** rMATS tables of retained introns (RI).

**Table S8:** Count tables from DEXSeq.

**Table S9:** Gene Ontology (GO) enrichment analysis results from ToppGene for the genes containing the 36 selected cassette exons (see also Fig. 3A). These 36 selected cassette exons were significantly differentially spliced (FDR < 1E-9 and difference in inclusion levels > 0.1) between hFK1 and hFK3 – the cell fractions representing the earliest and latest stages of kidney development - and were also found to show clear alternative splicing by manual inspection in the IGV genome browser.

**Table S10:** A list of RNA binding proteins and binding sites used for identifying putative splicing regulators.

**Program:** A compressed directory containing programs and datasets for data visualization.
