# Supplementary material for: Characterization of alternative mRNA splicing in cultured cell populations representing progressive stages of human fetal kidney development
Source: Sci Rep. 2022 Nov 15;12:19548. doi: 10.1038/s41598-022-24147-z (PMC9666651; doi:10.1038/s41598-022-24147-z)
Supplement: Supplementary file 2 — Supplementary Information 2. [file 41598_2022_24147_MOESM2_ESM.pdf]

Supplementary information for:
Characterization of alternative mRNA splicing in cultured cell populations representing progressive stages of human fetal kidney development

Yishay Wineberg[1,*], Itamar Kanter[1,*], Nissim Ben-Haim[1,*], Naomi Pode-Shakked[2,3,4,*], Efrat Bucris[1], Tali Hana Bar-Lev[1], Sarit Oriel[1], Harel Reinus[1], Yishai Yehuda[1], Rotem Gershon[2,3,4], Rachel Shukrun[2,4], Dekel Dov Bar-Lev[2,3], Achia Urbach[5], Benjamin Dekel[2,3,4,**], and Tomer Kalisky[1,6,**]

[1]*Department of Bioengineering and Bar-Ilan Institute of Nanotechnology and Advanced Materials (BINA), Bar-Ilan University, Ramat Gan, Israel 52900*
[2]*Pediatric Stem Cell Research Institute, Edmond and Lily Safra Children's Hospital, Sheba Medical Center, Tel-Hashomer, Israel 52621*
[3]*Division of Pediatric Nephrology, Sheba Medical Center, Tel-Hashomer, Israel 52621*
[4]*Sackler Faculty of Medicine, Tel-Aviv University, Tel-Aviv, Israel 69978*
[5]*The Mina and Everard Goodman Faculty of Life Sciences, Bar-Ilan University, Ramat-Gan, Israel 52900*
[*]*Co-first authors*
[**]*Co-senior authors*

[6]**Correspondence to:**
Tomer Kalisky
Department of Bioengineering, Bar-Ilan University, Ramat Gan, Israel 52900
Office: +972-3-738-4656
Email: tomer.kalisky@biu.ac.il

SUPPLEMENTARY TEXT

Manual calculation inclusion levels:

The gene FAT1 (Fig. S18) contains an alternatively skipped exon (SE) that we found in the literature but was not included in our gene model. For this exon we calculated the inclusion levels manually as follows. The inclusion level ψ of an alternatively spliced exon is defined as the fraction of transcripts that include the exon out of the total number of transcripts that either include the exon or skip over it [1,2]. This can be estimated from the density of reads that align to the upstream splice junction, the alternative exon itself, and the downstream splice junction, vs. the density of reads that align to the skipping splice junction that directly connects the upstream exon to the downstream exon. It is also possible to use only reads that span the splice junctions.

We define I to be the number of reads that map to the exon inclusion isoform and S to be the number of reads that map to the exon skipping isoform. Since we are interested in the density of reads, we need to normalize the read counts I and S by the "effective lengths" – the lengths of the isoform-specific segments that they align to. We therefore define l_I as the effective length of the exon-inclusion isoform, that is, the number of the unique positions to which reads can be aligned in the upstream splice junction, the alternative exon itself, and the downstream splice junction. Likewise we define l_S as the effective length of the exon-skipping isoform, that is, the number of unique positions to which reads can be aligned in the skipping splice junction that directly connects the upstream exon to the downstream exon (see Fig. S1 of [1] and supplementary note of [2]).

Given the effective lengths l_I and l_S for each isoform, the inclusion level ψ can now be estimated from read counts I and S as:

$$\hat{\psi} = \frac{(I/l_I)}{(I/l_I) + (S/l_S)}$$

For our manual calculation we counted only reads that span the splice junctions, and approximated the effective lengths as the number of junctions from which each isoform is composed (see Fig. S1 of [1]), that is, for a **skipped exon**: $l_I = 2$ and $l_S = 1$ - two splice junctions for the inclusion isoform and one splice junction for the skipping isoform.

Quantifying alternative splicing using DEXSeq:

For some of the genes, we also used DEXSeq [3] to validate differential expression in specific exons by counting the number of reads that align to each exon (or segment of an exon) within each cell population. In order to take into account the different depths of each transcriptome due to the varying number of cells in each population, we normalized the exon-specific counts by dividing by the total number of reads that align to the specific gene.

DEXSeq was used either to complement rMATS (e.g. for the mutually exclusive exons in FGFR2, Fig. S36) or to quantify alternative splicing of types that are not covered by rMATS (e.g. for the alternative 3' ends in the gene EPB41L5, Fig. S42).

Supplementary figures

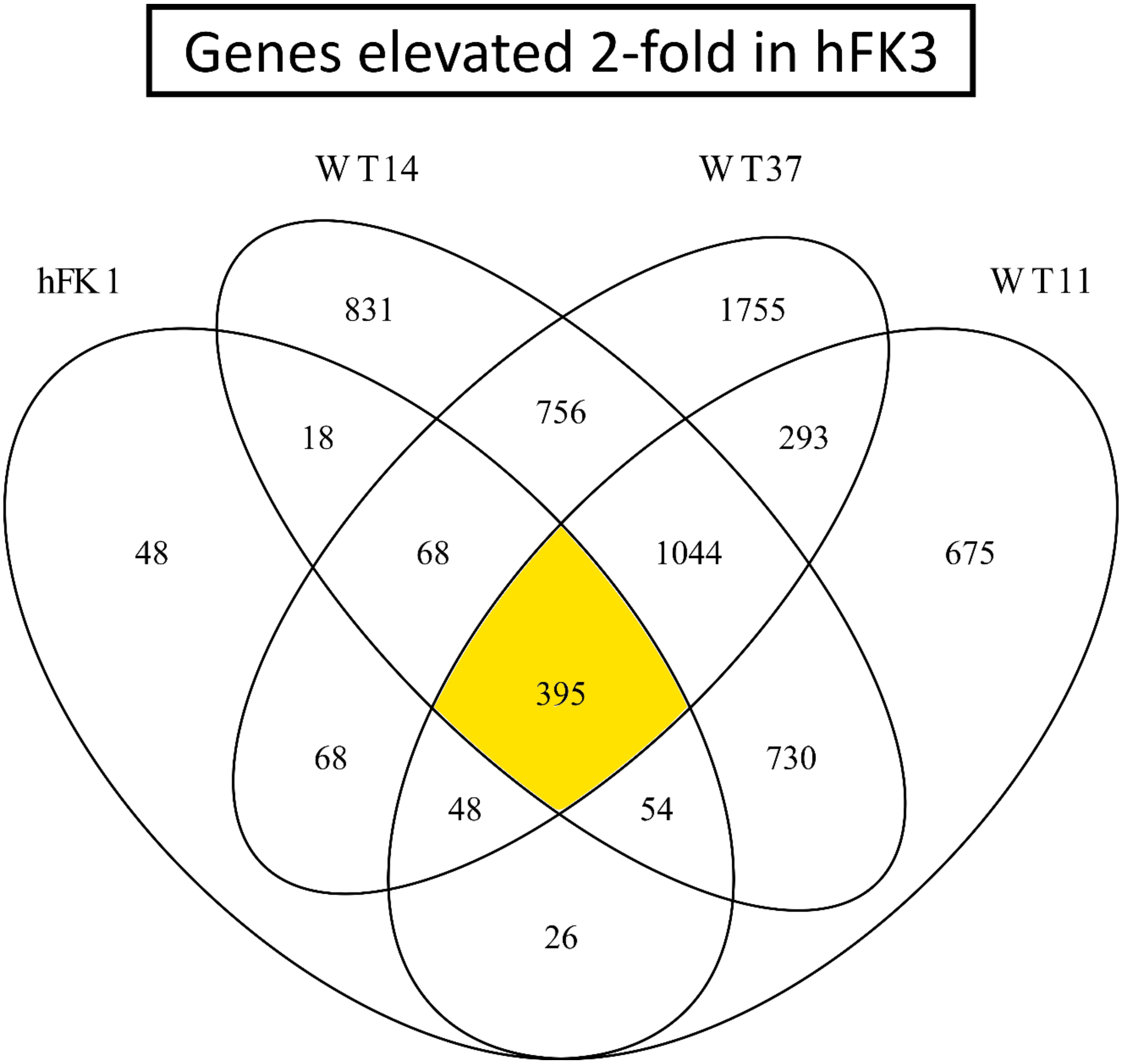

Figure S1: We selected a set of 395 genes that were found by intersecting all the gene sets that were upregulated at least 2-fold (log2foldChange > 1) in hFK3 (the mature fetal developmental fraction) with respect to hFK1, WT11, WT14, and WT37, and found that they are related to epithelial differentiation (Fig. 2D and Table S2). Shown is a Venn diagram of differentially expressed genes with log2foldChange > 1. Each oval represents a set of genes that were found to be upregulated in hFK3 with respect to the labeled cell fraction (hFK1, WT11, WT14, or WT37). We note that the enrichment that we observed was quite robust and did not depend much on the exact parameters for choosing these genes (e.g. the exact threshold for log2foldchange etc.)

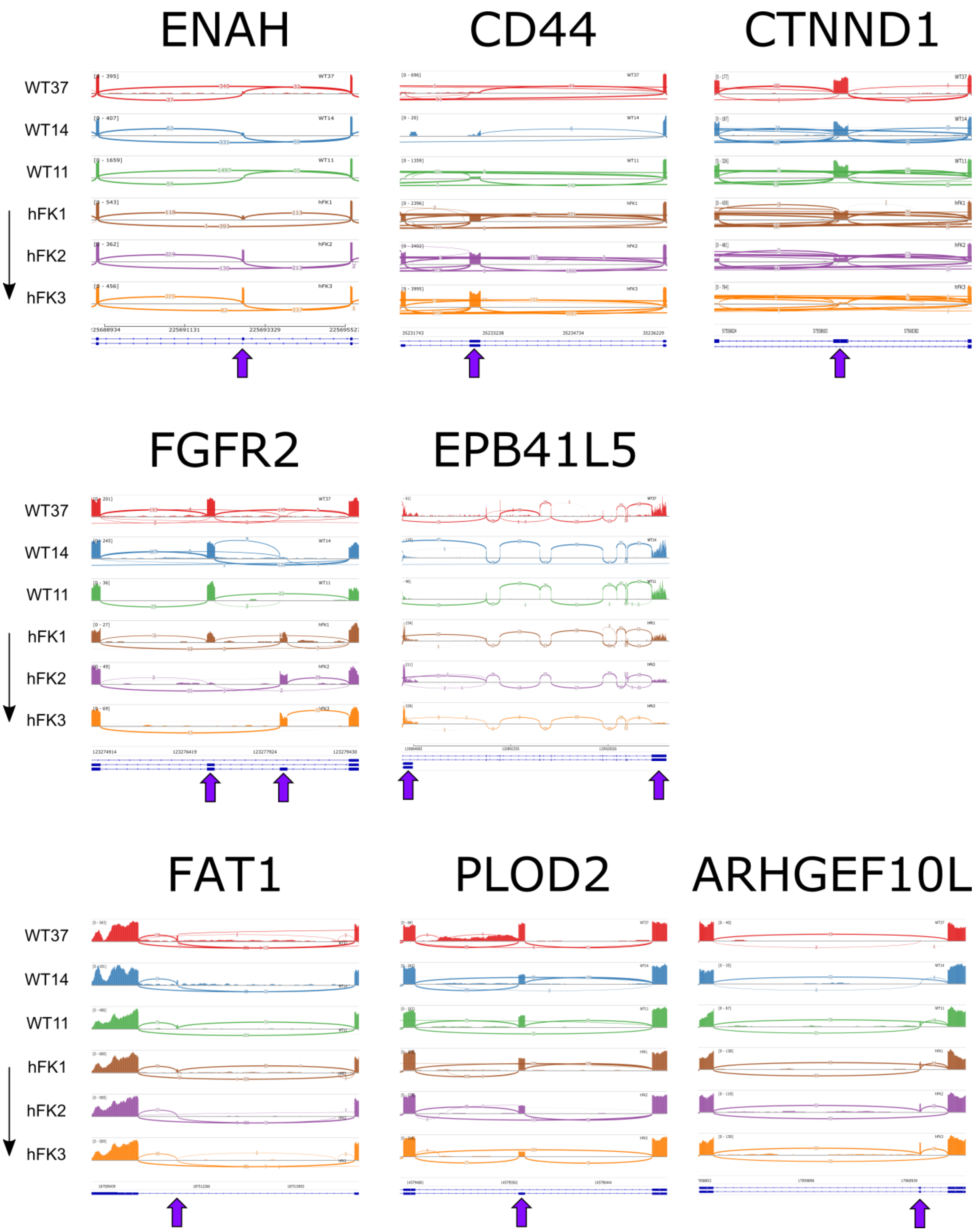

Figure S2: Cells at the early stage of human kidney development (hFK1) have a mesenchymal splice-isoform profile that is similar to that observed in blastemal-predominant Wilms' tumor patient-derived xenografts (WT37, WT14, and WT11). Shown are Sashimi plots for the genes ENAH [4–6], CD44 [7,8], CTNND1 [6,9], FGFR2 [9,10], EPB41L5 [6,11], FAT1 [4,12], PLOD2 [4,13,14], and ARHGEF10L [15–17]. These genes were previously shown to be alternatively spliced in mesenchymal and epithelial

tissues. The genes ENAH and CD44 contain cassette exons that are low in the Wilms' tumor xenografts and increase during kidney development, whereas CTNND1 contains a cassette exon that is high in Wilms' tumors and decreases during kidney development. FGFR2 contains two mutually exclusive exons, one of which is high in Wilms' tumors and decreases during kidney development, and the other which is low in Wilms' tumors and increases during kidney development. Likewise, EPB41L5 has two isoforms with alternative 3’ ends, one of which is predominant in the Wilms’ tumor xenografts and decreases during kidney development, and the other which is low in Wilms' tumors and increases during kidney development. FAT1 and PLOD2, two genes for which alternative splicing was previously found to be regulated by the RNA binding protein RBFOX2 [4], contain cassette exons that are highly expressed in Wilms' tumors and decrease during kidney development, while ARHGEF10L, a gene for which alternative splicing was previously found to be regulated by the RNA binding proteins ESRP1 and ESRP2 [15], contains a cassette exon that is low in Wilms' tumors and increases during kidney development.

Correlation between gene expression and inclusion levels

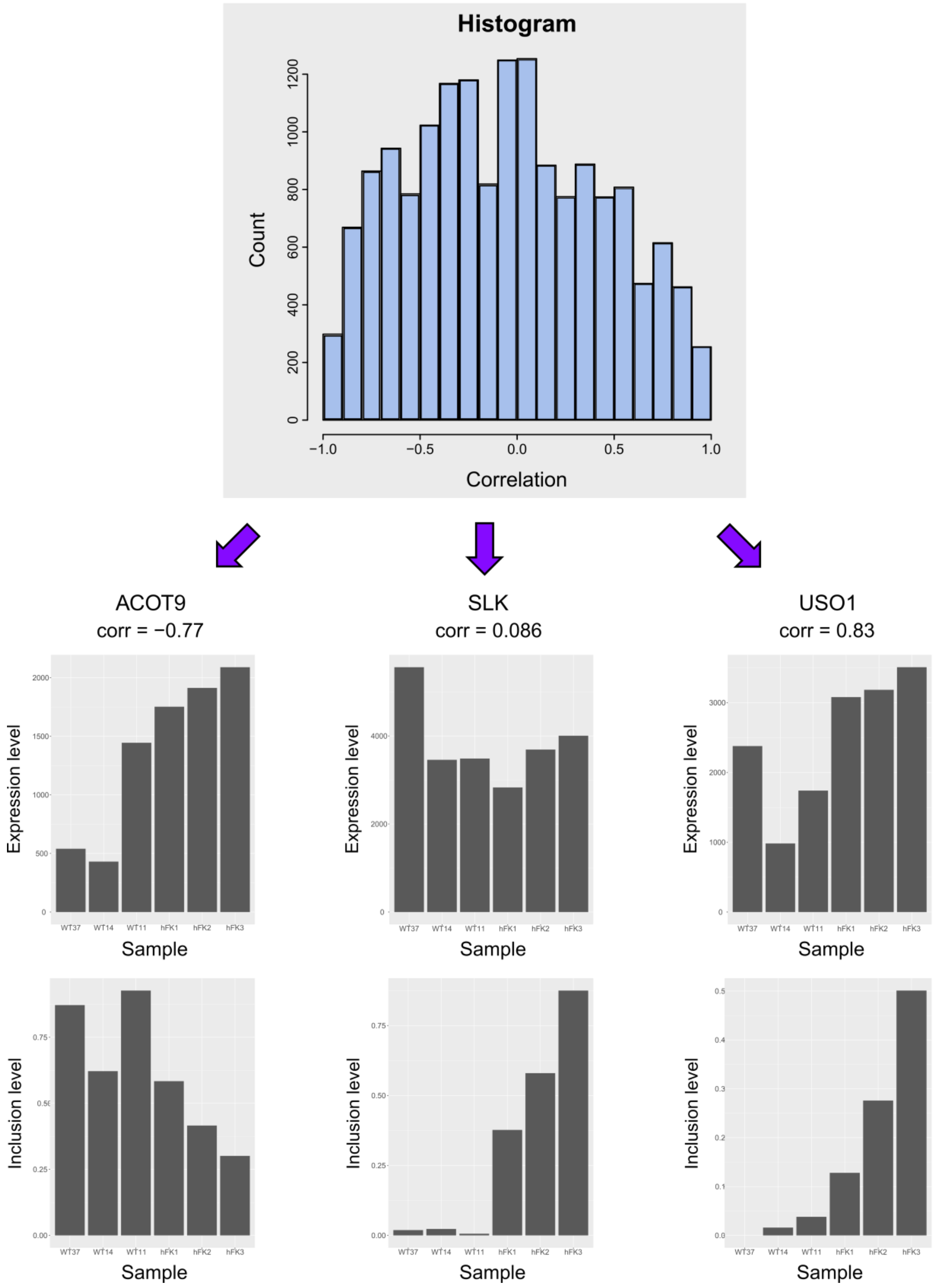

Figure S3: No consistent relationship between gene expression levels and exon inclusion levels. Shown is a histogram of correlation values between expression levels and cassette exon inclusion levels within the same gene, as well as specific examples. It can be seen that for some genes expression and splicing are strongly correlated (USO1) or anti-correlated (ACOT9) while for others they are not (SLK).

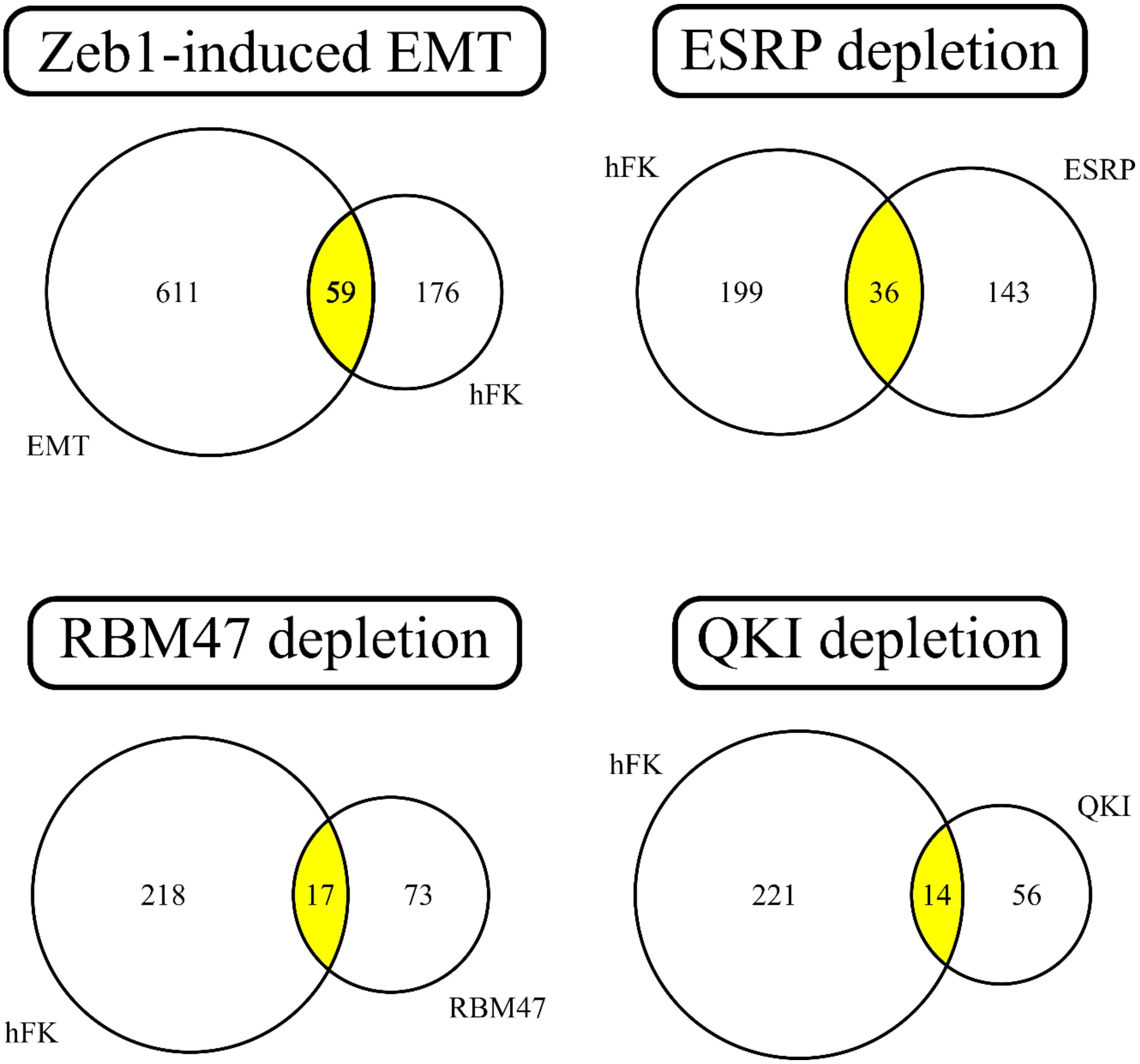

Figure S4: A comparison of cassette exons that were found in the present study to be alternatively spliced between cell fractions representing early (hFK1) and late (hFK3) stages of human kidney development vs. alternatively spliced cassette exons that were previously observed by Yang *et al.* [14] in cells from a human H358 epithelial non-small cell lung cancer (NSCLC) cell line undergoing Epithelial to Mesenchymal Transition (EMT). Shown are Venn diagrams comparing the findings of our study ("hFK") to four different experiments performed in Yang *et al.* [14]: (1) Zeb1-induced EMT, (2) ESRP1/2 depletion, (3) RBM47 depletion, and (4) QKI depletion. In order to perform a consistent comparison, we followed the selection criteria of Yang *et al.* [14] and selected cassette exons for which the FDR < 0.05 and the absolute difference in inclusion levels > 0.05. For example, we found that the cassette exons located within the genes CD44 and ENAH are common to both our experiments (hFK) and also to the Zeb1-induced EMT, ESRP1/2 depletion, and RBM47 depletion experiments.

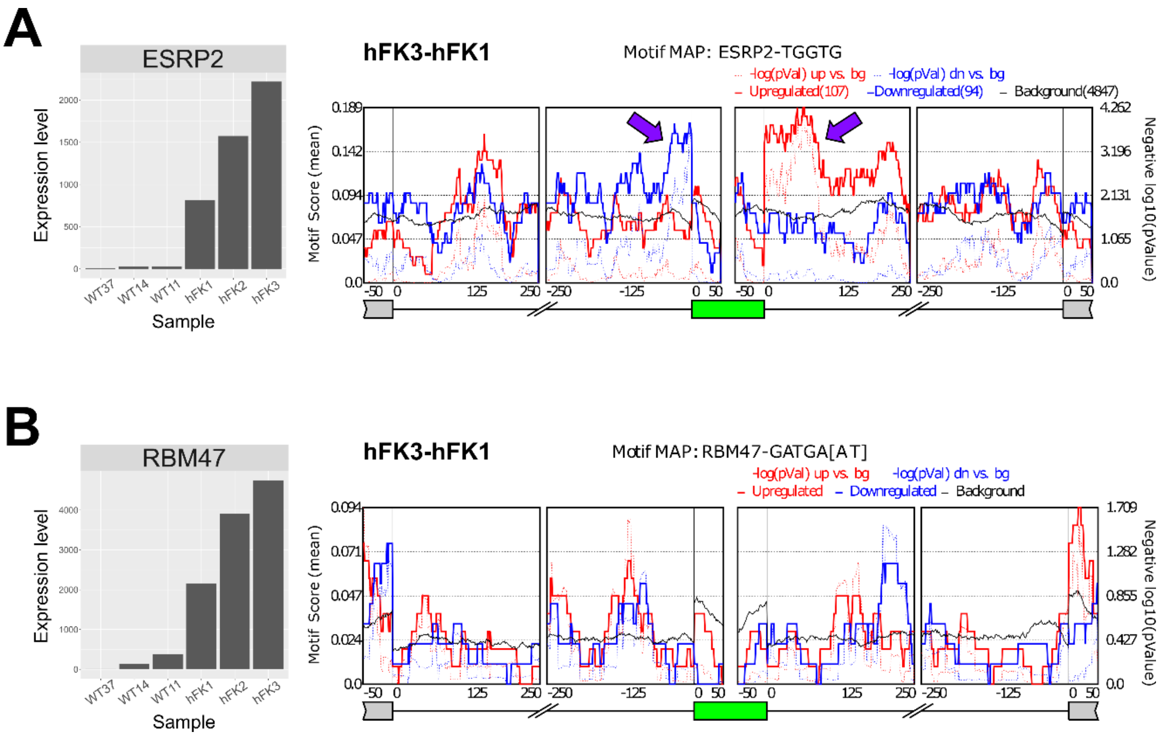

Figure S5: RNA binding motif enrichment analysis indicates that the mRNA binding protein ESRP2 regulates alternative mRNA splicing during human kidney development. (A) ESRP2 expression levels are low in all Wilms' tumor samples and monotonically increase along human kidney development, starting with moderate levels in hFK1 and reaching a maximum in hFK3. Likewise, exons that were enhanced in hFK3 (with respect to hFK1) are enriched for ESRP2 binding sites at their downstream 3' flanking introns (red curve), while exons that are silenced in hFK3 are enriched for ESRP2 binding sites at their upstream 5' flanking introns (blue curve). (B) Although the RNA binding protein RBM47 [14] is also differentially over-expressed in hFK3 vs. hFK1, we did not find significant motif enrichment for this gene.

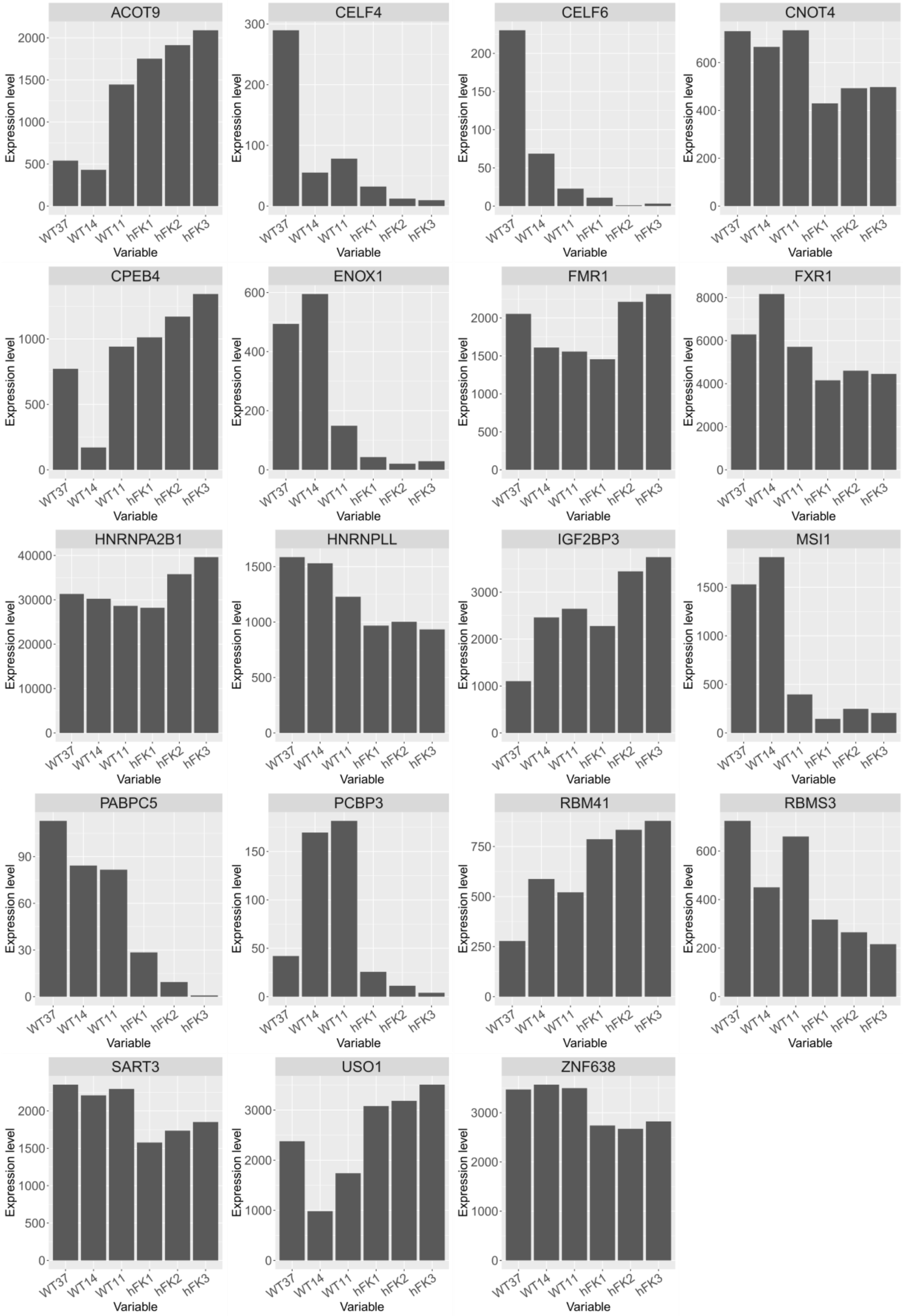

Figure S6: Additional RNA binding proteins that are differentially expressed between the cell fractions representing the early (hFK1) and late (hFK3) stages of human kidney development or between the blastemal-predominant Wilms' tumor patient-derived

xenografts (WT37, WT14, and WT11) and hFK3. These RBP's might also be involved in splicing regulation in the developing fetal kidney or in the development of Wilms' tumors, but we did not find any significant motif enrichment for these genes.

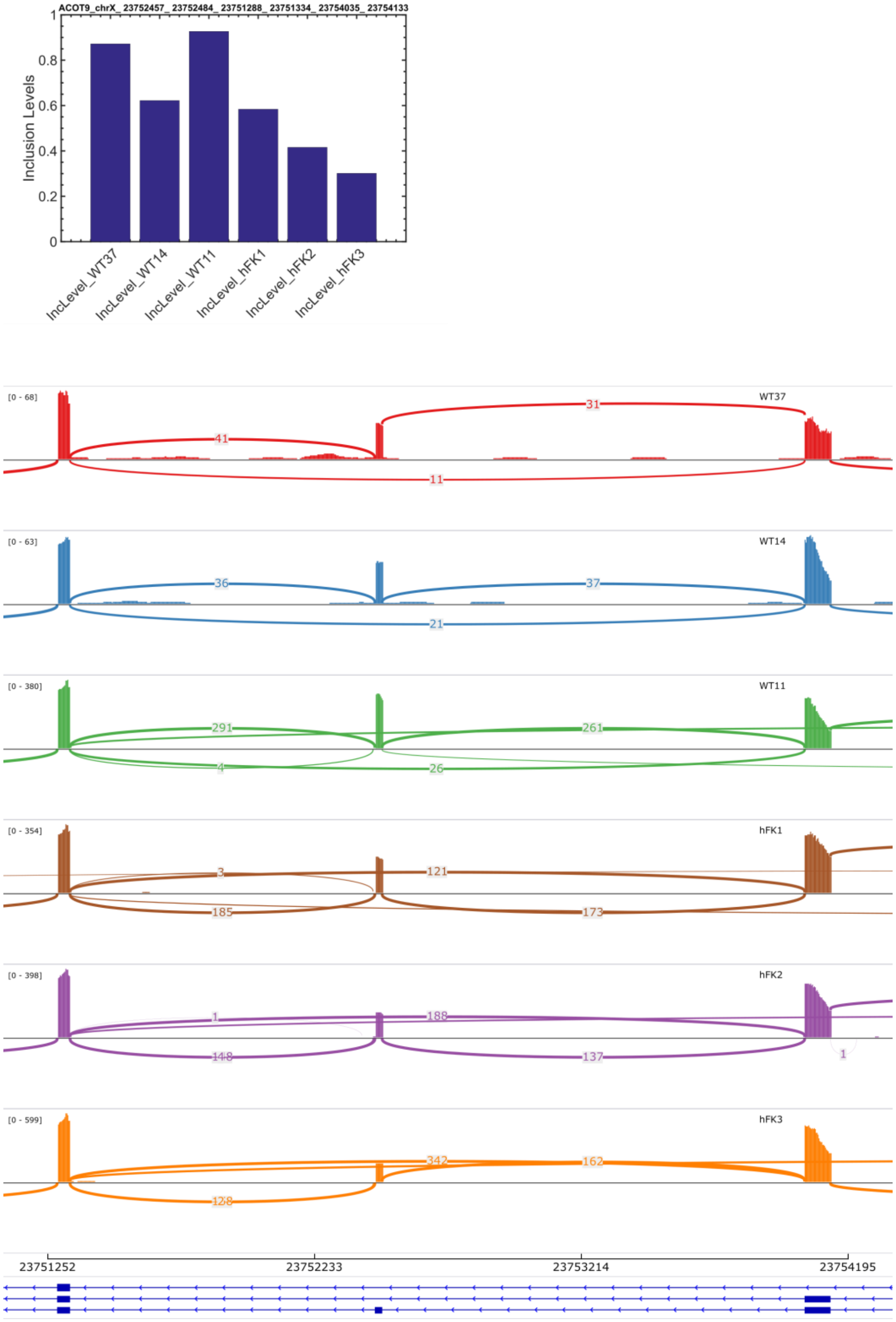

Figure S7: The gene ACOT9 contains a cassette exon (skipped exon, SE) that is highly expressed in the Wilms' tumor xenografts (WT37, WT14, and WT11) and the cells representing the early stage of human kidney development (hFK1), and whose expression gradually decreases during later stages of kidney development (hFK2 and hFK3). Shown are a Sashimi plot and a barplot of inclusion levels obtained from rMATS.

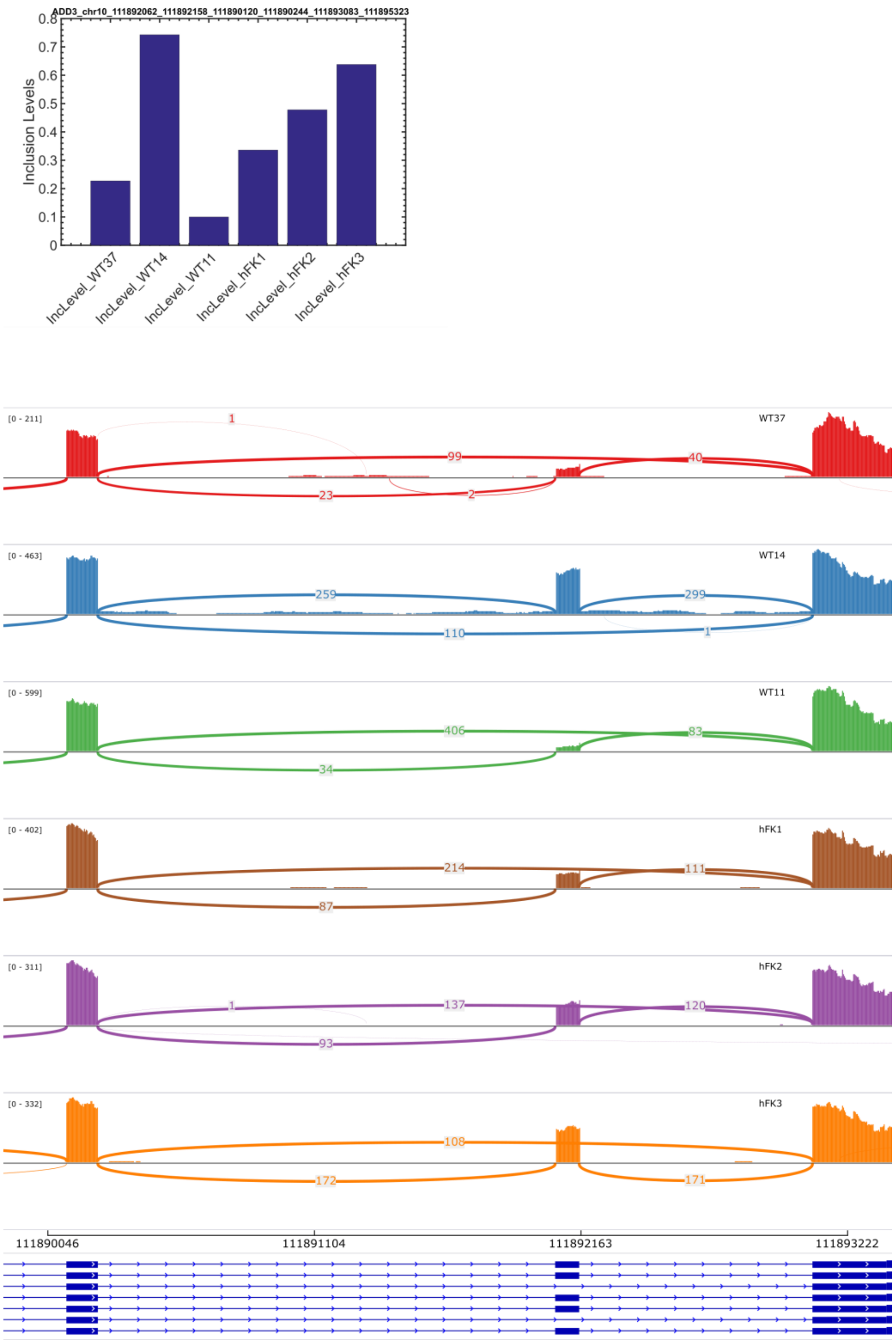

Figure S8: The gene ADD3 contains a cassette exon (skipped exon, SE) that is lowly expressed in two out of three of the Wilms' tumor xenografts (WT37 and WT11) and the cells representing the early stage of human kidney development (hFK1), and whose expression gradually increases during later stages of kidney development (hFK2 and hFK3). Shown are a Sashimi plot and a barplot of inclusion levels obtained from rMATS.

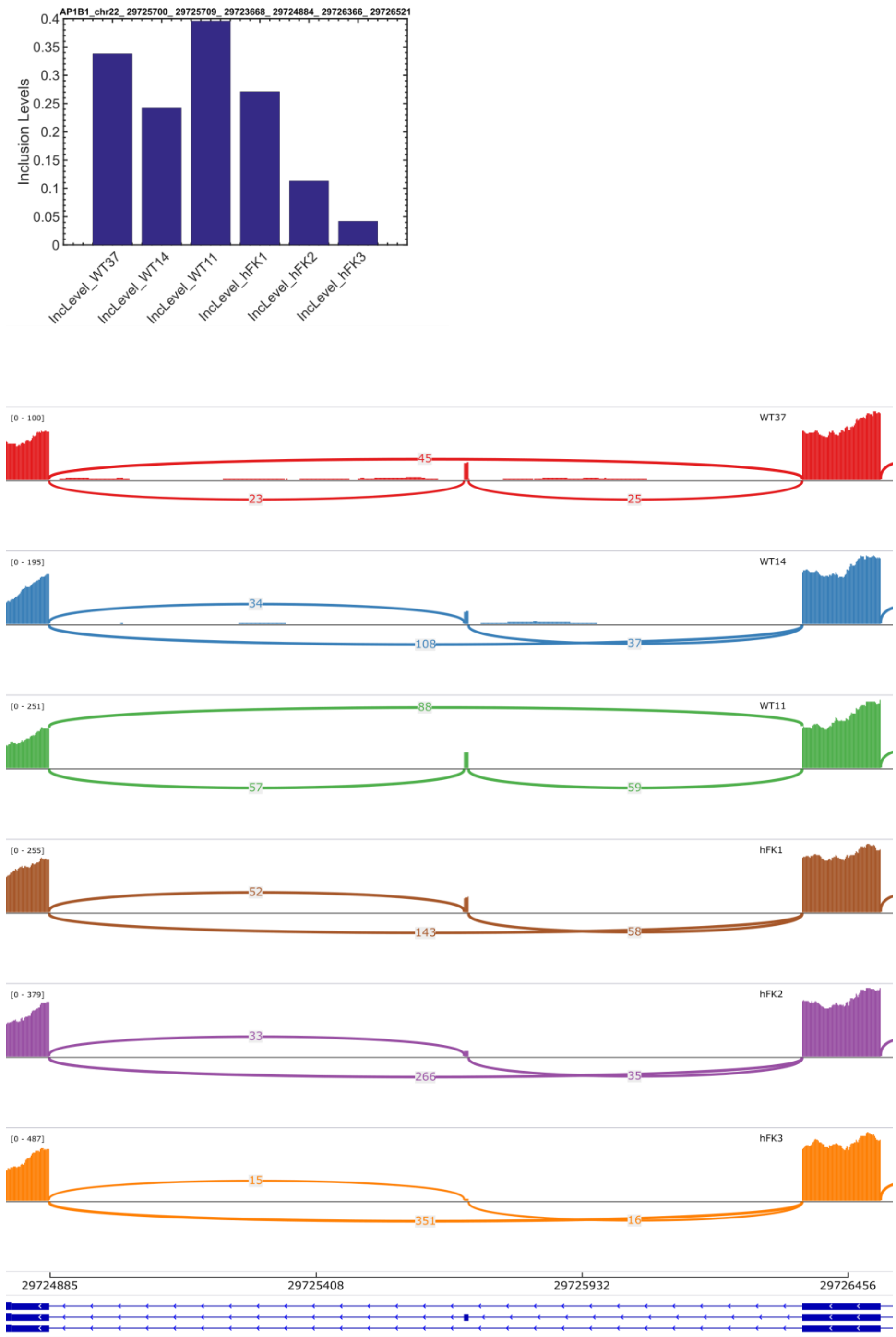

Figure S9: The gene AP1B1 contains a cassette exon (skipped exon, SE) that is highly expressed in the Wilms' tumor xenografts (WT37, WT14, and WT11) and the cells representing the early stage of human kidney development (hFK1), and whose expression gradually decreases during later stages of kidney development (hFK2 and hFK3). Shown are a Sashimi plot and a barplot of inclusion levels obtained from rMATS.

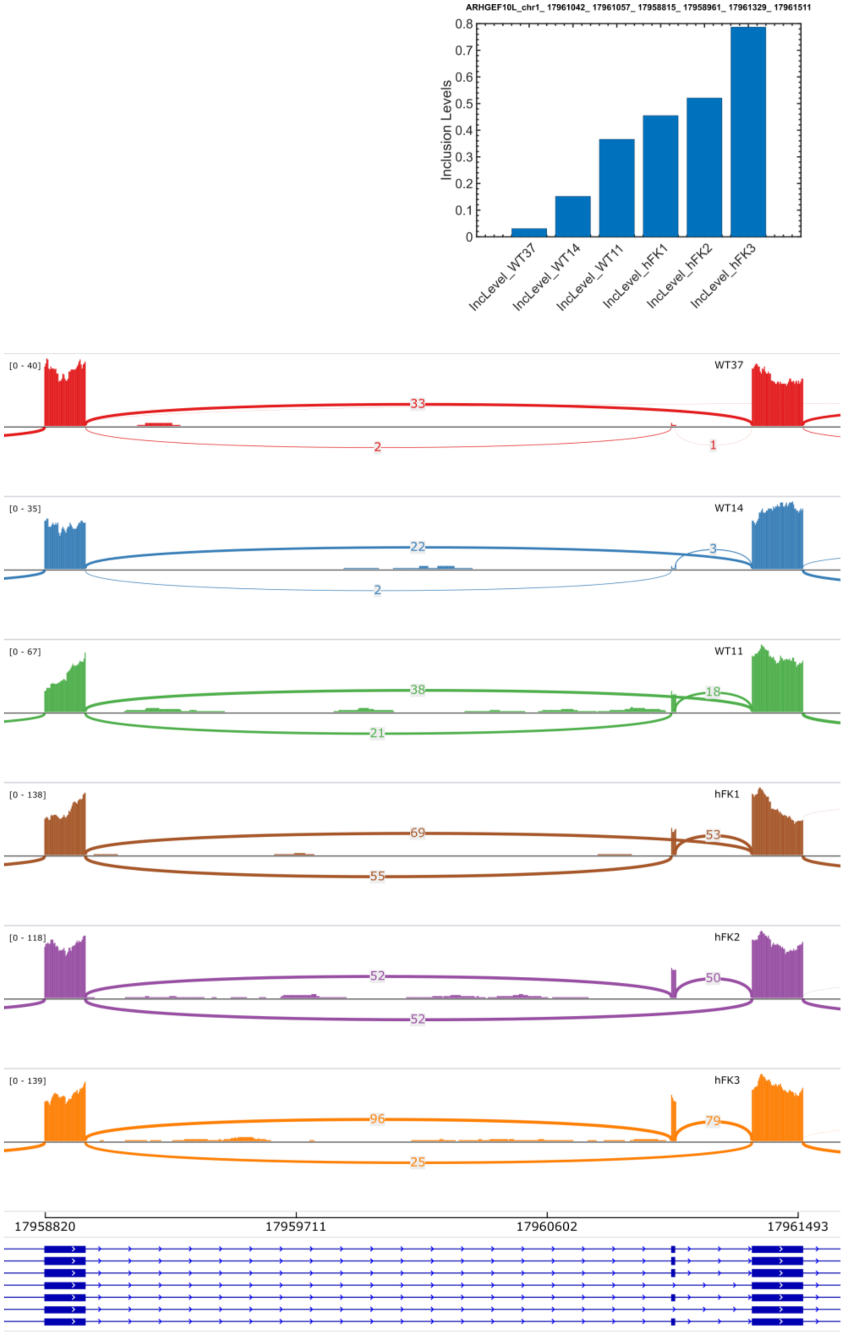

Figure S10: The gene ARHGEF10L [15–17], a gene for which alternative splicing was previously found to be regulated by the RNA binding proteins ESRP1 and ESRP2 [15], contains a cassette exon (skipped exon, SE) that is lowly expressed in the Wilms' tumor xenografts (WT37, WT14, and WT11) and the cells representing the early stage of human kidney development (hFK1), and whose expression gradually increases during later stages of kidney development (hFK2 and hFK3). Shown are a Sashimi plot and a barplot of inclusion levels obtained from rMATS.

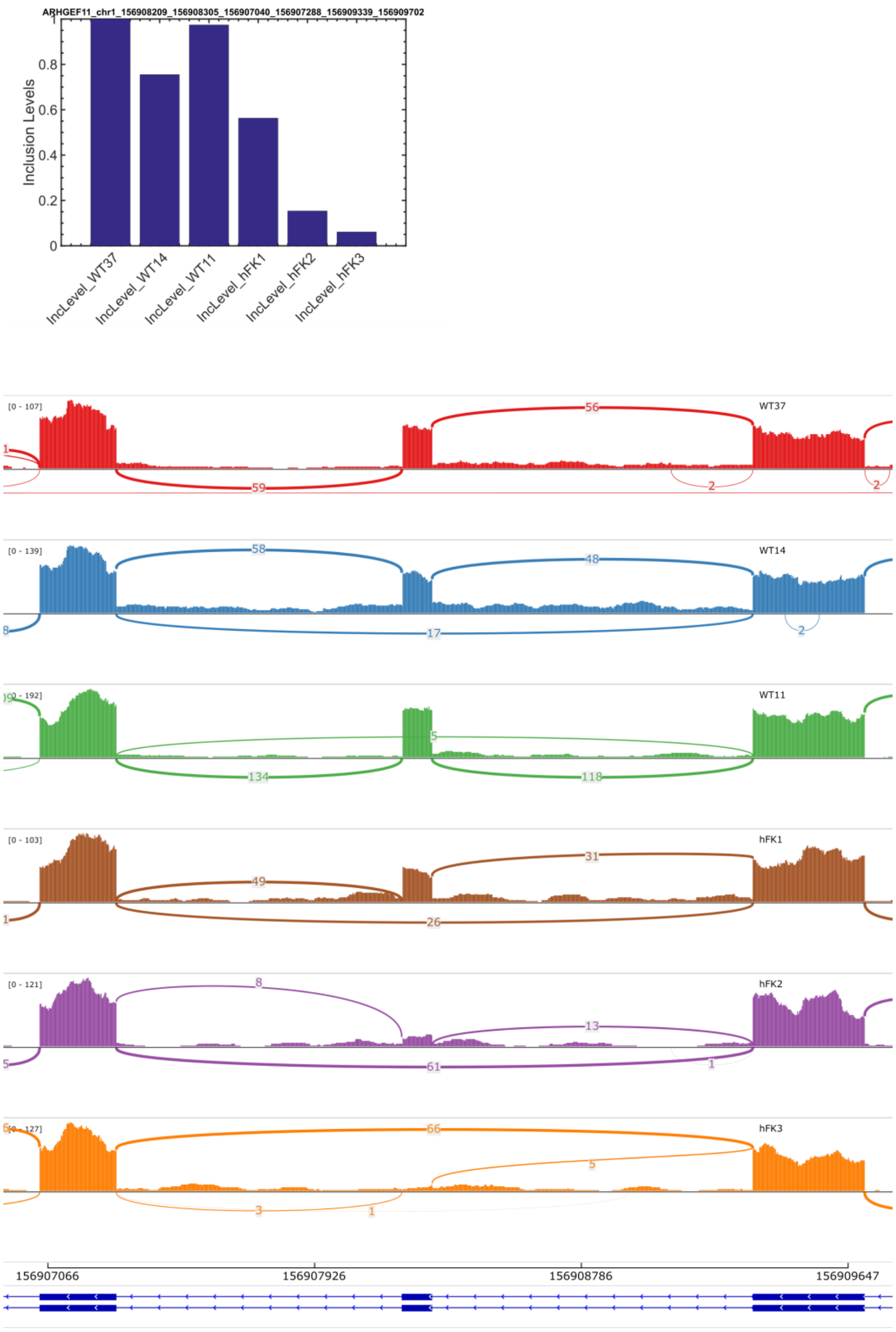

Figure S11: The gene ARHGEF11 contains a cassette exon (skipped exon, SE) that is highly expressed in the Wilms' tumor xenografts (WT37, WT14, and WT11) and the cells representing the early stage of human kidney development (hFK1), and whose expression gradually decreases during later stages of kidney development (hFK2 and hFK3). Shown are a Sashimi plot and a barplot of inclusion levels obtained from rMATS.

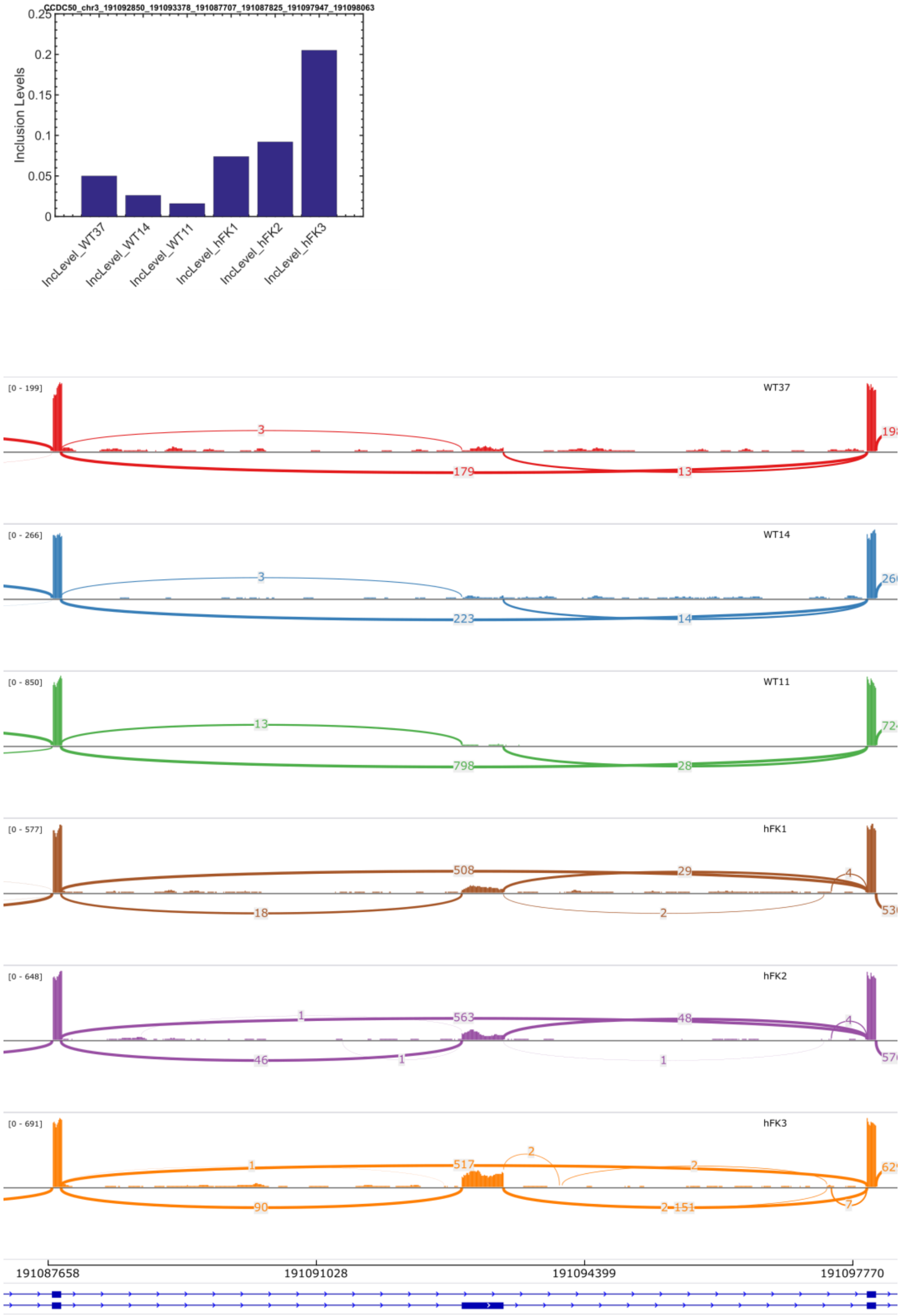

Figure S12: The gene CCDC50 contains a cassette exon (skipped exon, SE) that is lowly expressed in the Wilms' tumor xenografts (WT37, WT14, and WT11) and the cells representing the early stage of human kidney development (hFK1), and whose expression gradually increases during later stages of kidney development (hFK2 and hFK3). Shown are a Sashimi plot and a barplot of inclusion levels obtained from rMATS.

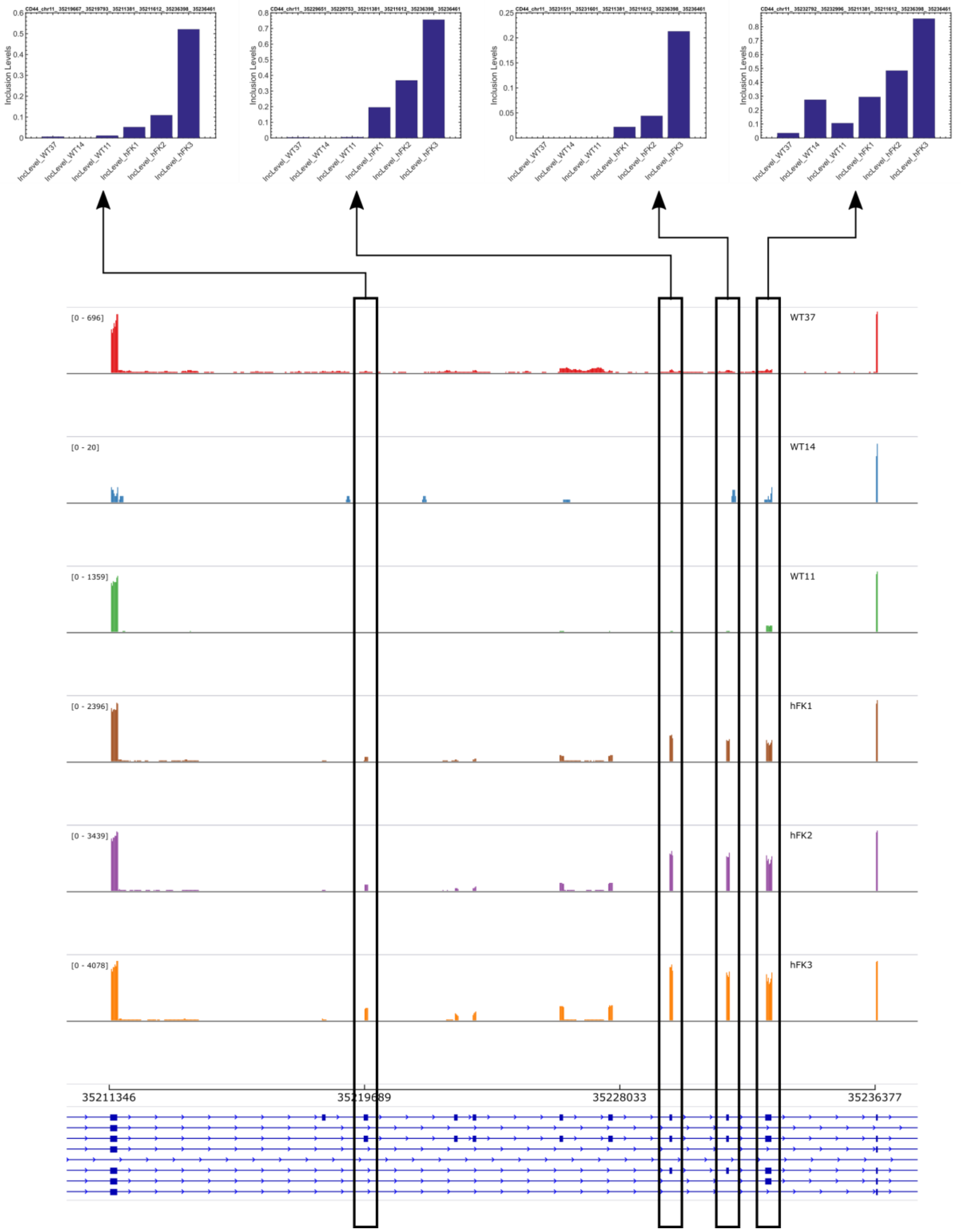

Figure S13: The gene CD44 [7,8], that was previously shown to be alternatively spliced between mesenchymal and epithelial tissues, contains cassette exons (skipped exons, SE) that are lowly expressed in the Wilms' tumor xenografts (WT37, WT14, and WT11) and the cells representing the early stage of human kidney development (hFK1), and whose expression gradually increases during later stages of kidney development (hFK2 and hFK3). Shown are a Sashimi plot and barplots of inclusion levels obtained from rMATS.

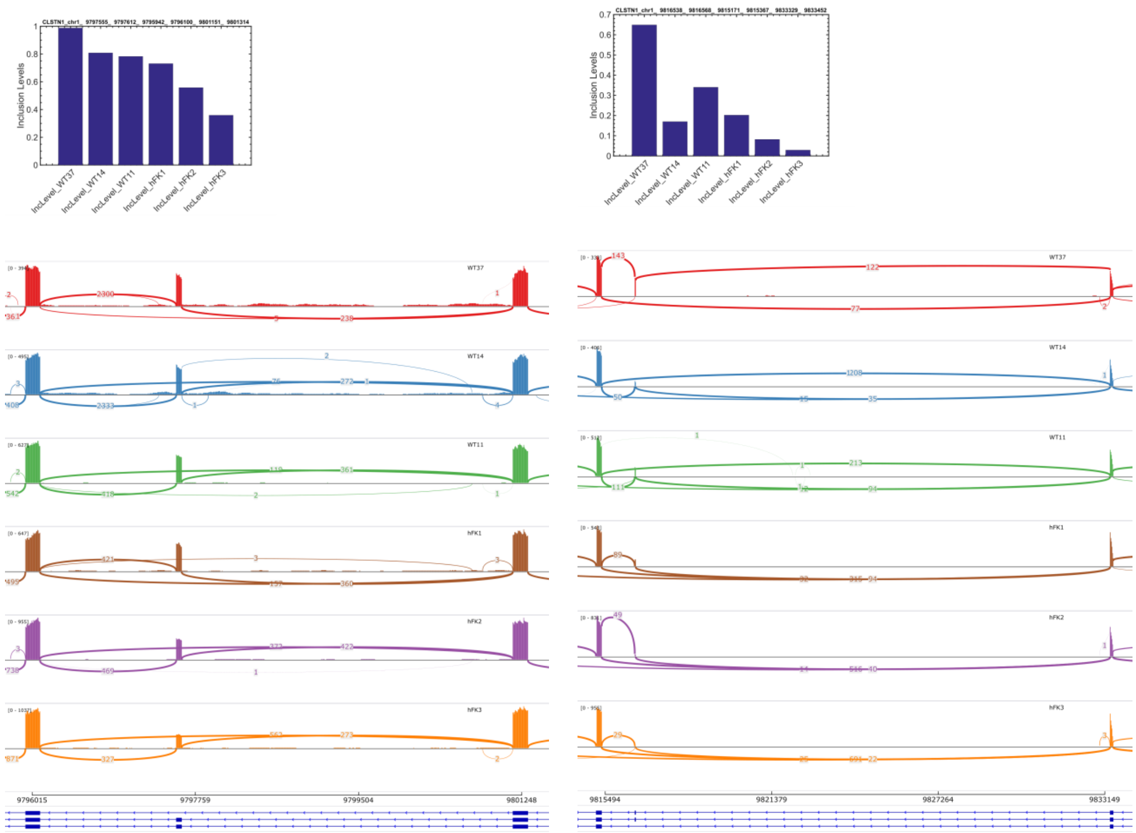

Figure S14: The gene CLSTN1 contains cassette exons (skipped exons, SE) that are highly expressed in the Wilms' tumor xenografts (WT37, WT14, and WT11) and the cells representing the early stage of human kidney development (hFK1), and whose expression gradually decreases during later stages of kidney development (hFK2 and hFK3). Shown are Sashimi plots and barplots of inclusion levels obtained from rMATS.

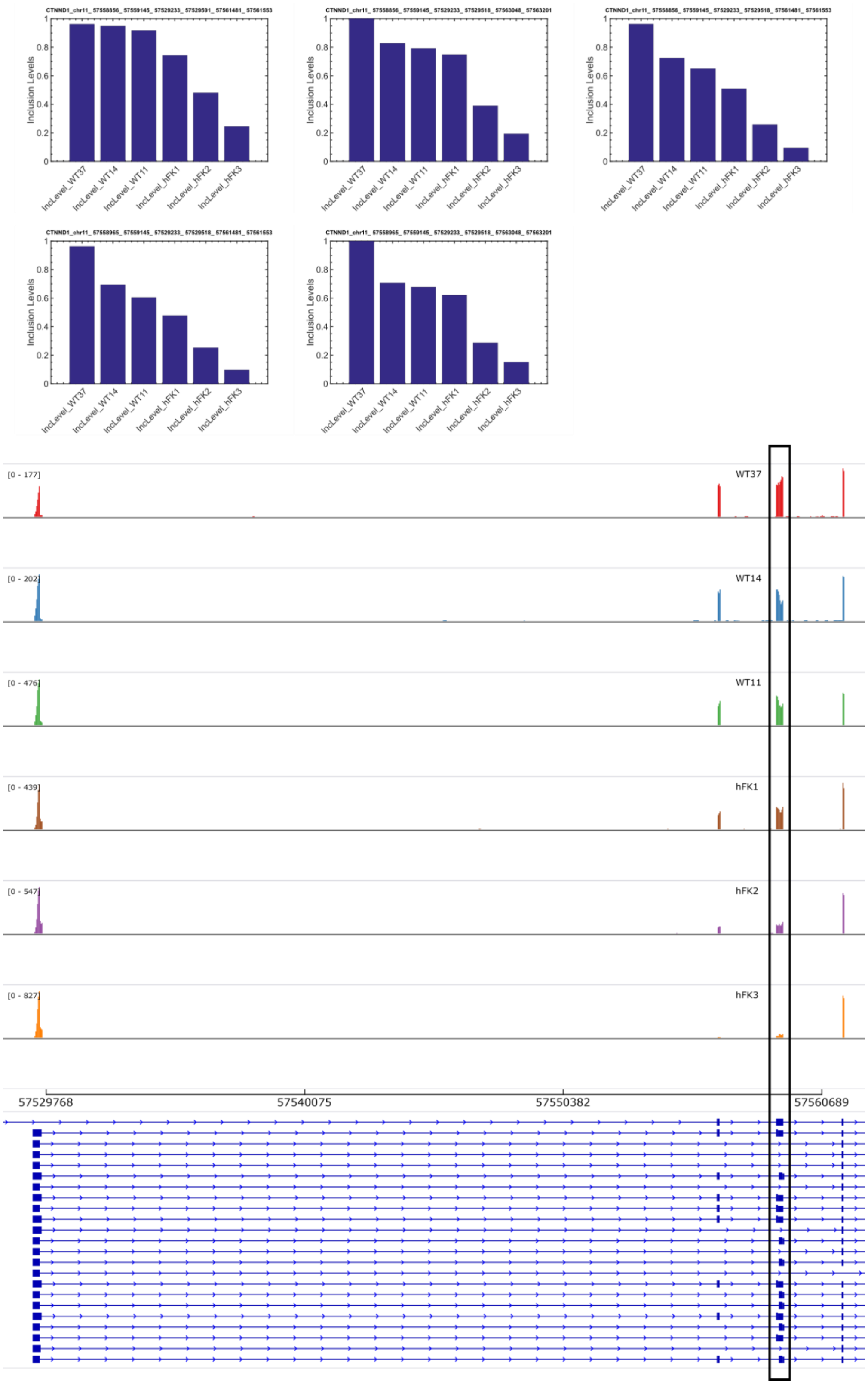

Figure S15: the gene CTNND1 [6,9], that was previously shown to be alternatively spliced between mesenchymal and epithelial tissues, contains a cassette exon (skipped

exon, SE) that is highly expressed in the Wilms' tumor xenografts (WT37, WT14, and WT11) and the cells representing the early stage of human kidney development (hFK1), and whose expression gradually decreases during later stages of kidney development (hFK2 and hFK3). Shown are a Sashimi plot and barplots of inclusion levels obtained from rMATS. Note that this exon is composed of two parts.

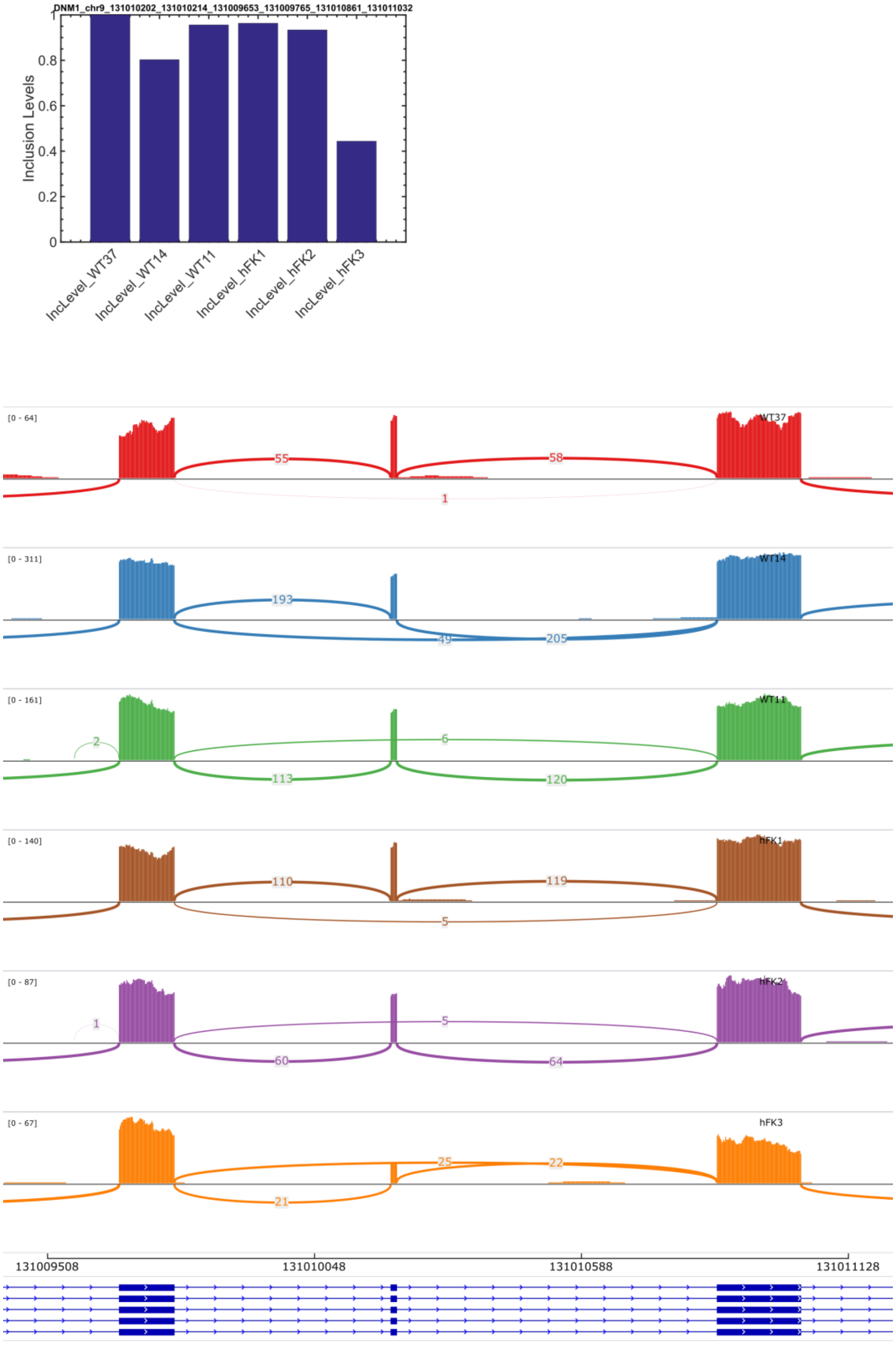

Figure S16: The gene DNM1 contains a cassette exon (skipped exon, SE) that is highly expressed in the Wilms' tumor xenografts (WT37, WT14, and WT11) and the cells representing the early and intermediate stages of human kidney development (hFK1

and hFK2), and whose expression decreases during the late stage of kidney development (hFK3). Shown are a Sashimi plot and a barplot of inclusion levels obtained from rMATS.

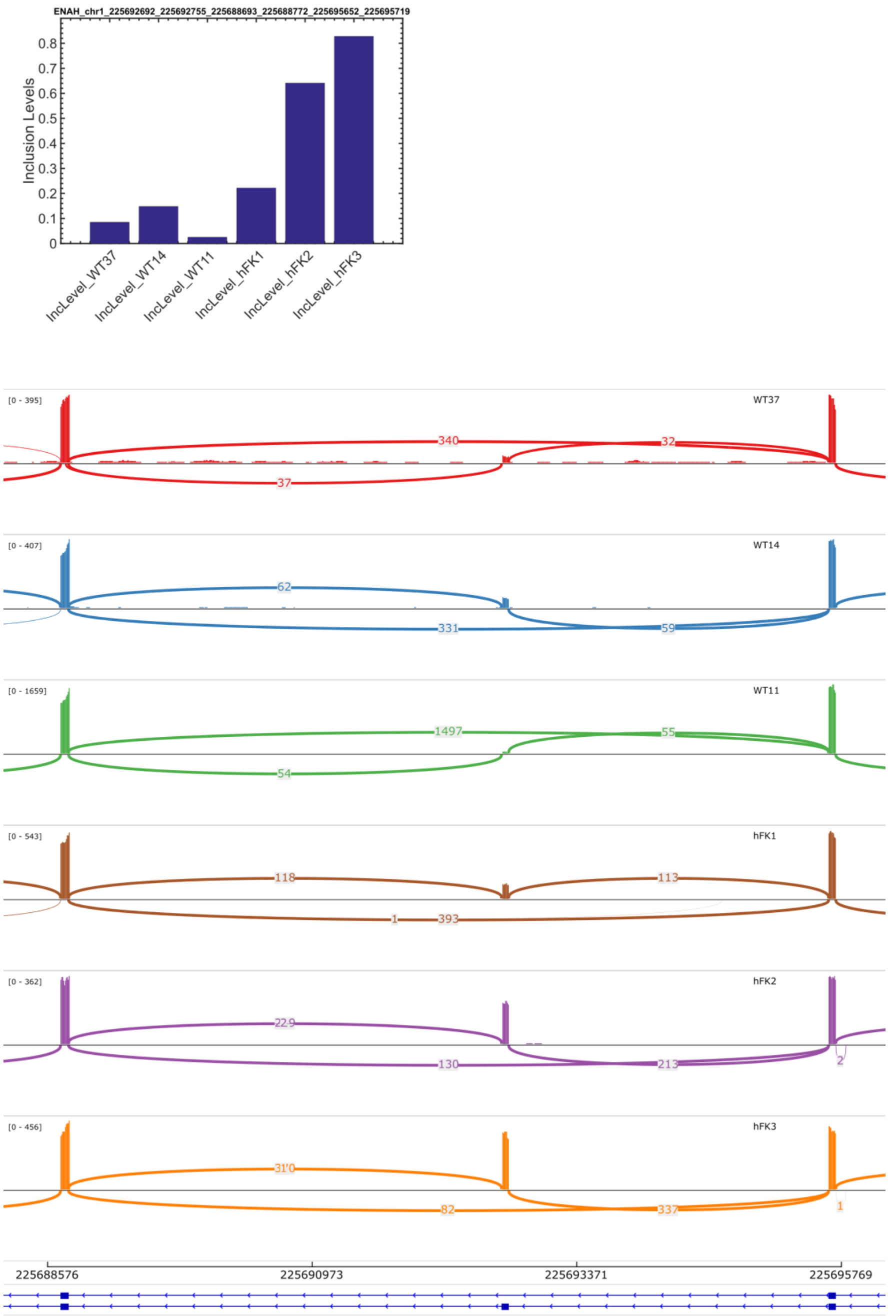

Figure S17: The gene ENAH [4–6], that was previously shown to be alternatively spliced between mesenchymal and epithelial tissues, contains a cassette exon (skipped exon, SE) that is lowly expressed in the Wilms' tumor xenografts (WT37, WT14, and WT11) and the cells representing the early stage of human kidney development (hFK1), and whose expression gradually increases during later stages of kidney development (hFK2 and hFK3). Shown are a Sashimi plot and a barplot of inclusion levels obtained from rMATS.

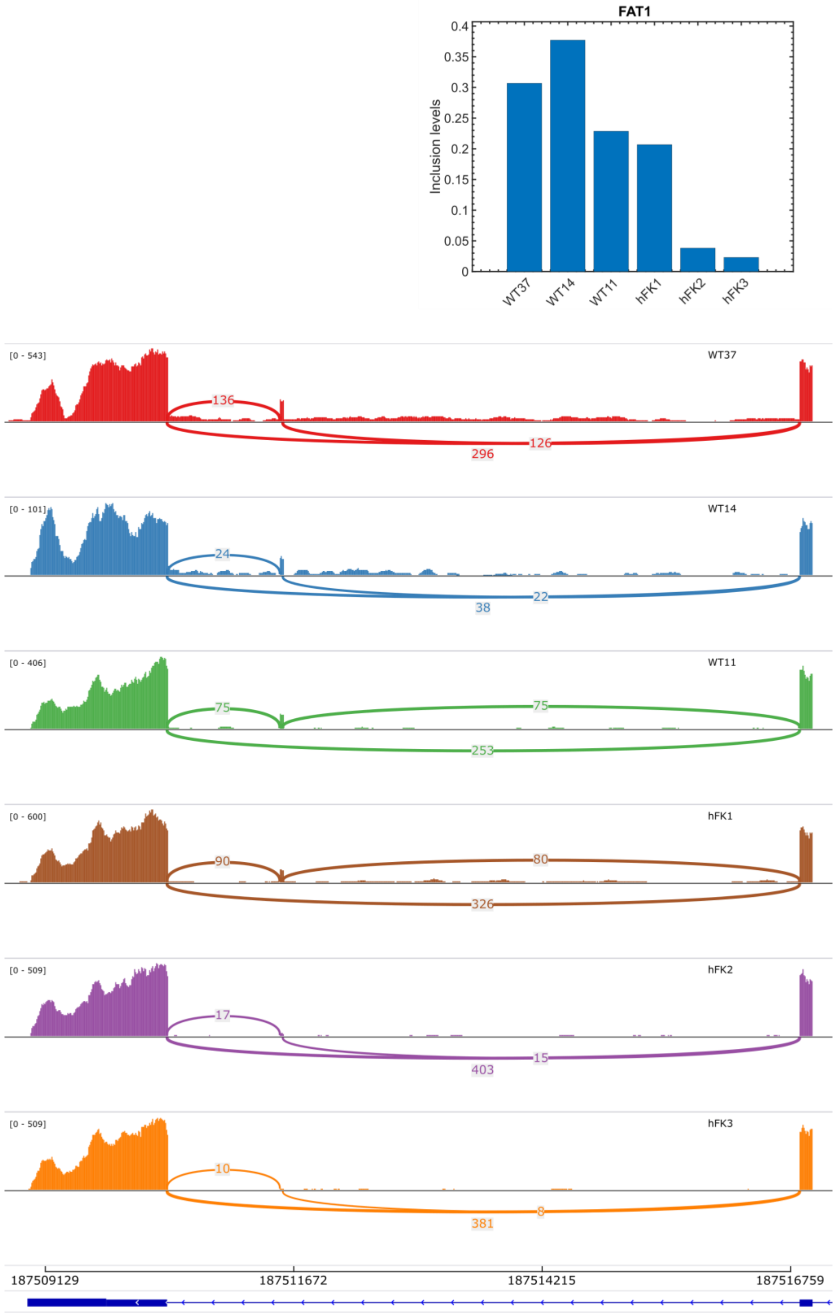

Figure S18: The gene FAT1 [4,12], a gene for which alternative splicing was previously found to be regulated by the RNA binding protein RBFOX2 [4], contains a cassette exon (skipped exon, SE) that is highly expressed in the Wilms' tumor xenografts (WT37, WT14, and WT11) and the cells representing the early stage of human kidney development (hFK1), and whose expression gradually decreases during later stages of kidney development (hFK2 and hFK3). Shown are a Sashimi plot and a barplot of inclusion levels that were manually calculated.

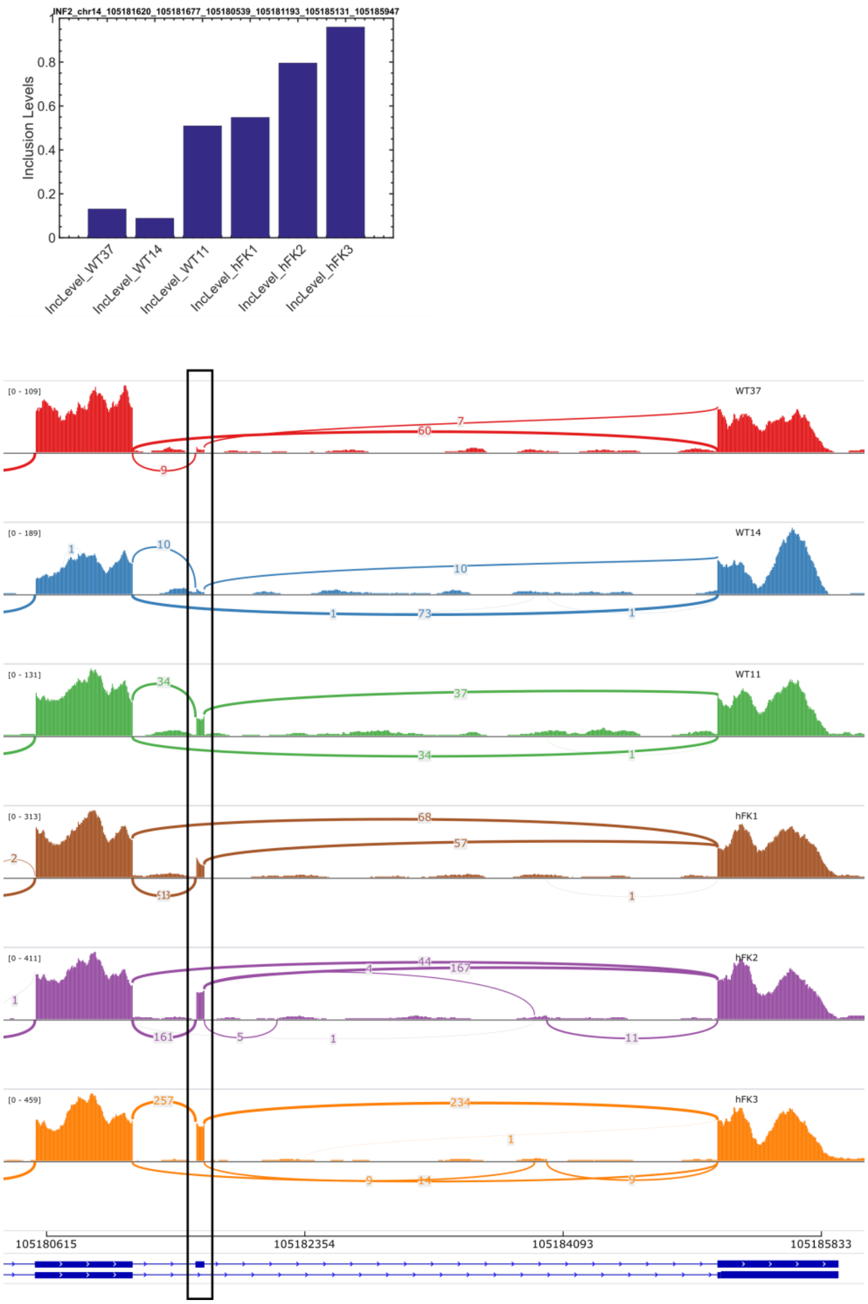

Figure S19: The gene INF2 contains a cassette exon (skipped exon, SE) that is lowly expressed in the Wilms' tumor xenografts (WT37, WT14, and WT11) and the cells representing the early stage of human kidney development (hFK1), and whose expression gradually increases during later stages of kidney development (hFK2 and hFK3). Shown are a Sashimi plot and a barplot of inclusion levels obtained from rMATS.

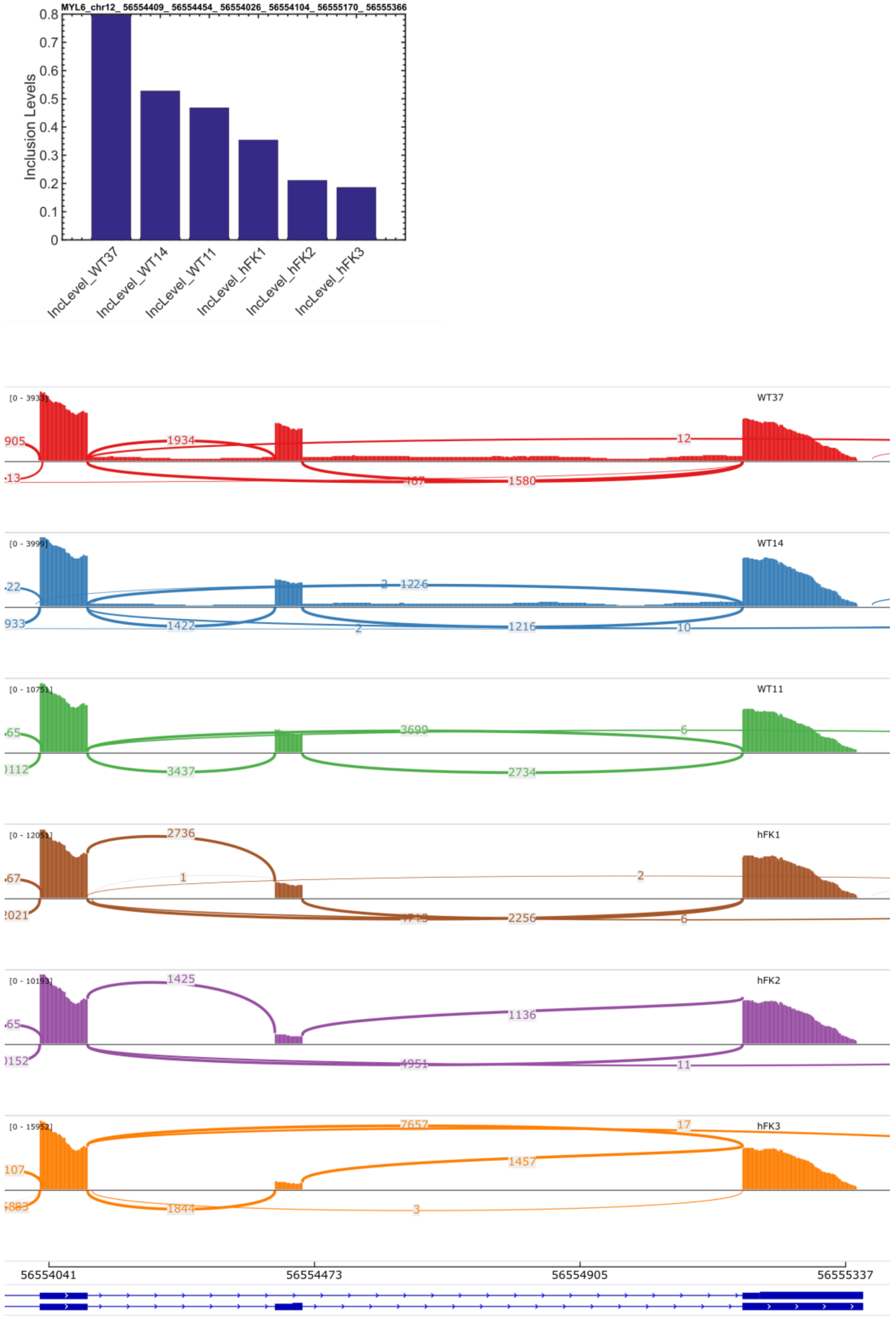

Figure S20: The gene MYL6 contains a cassette exon (skipped exon, SE) that is highly expressed in the Wilms' tumor xenografts (WT37, WT14, and WT11) and the cells representing the early stage of human kidney development (hFK1), and whose expression decreases during later stages of kidney development (hFK2 and hFK3). Shown are a Sashimi plot and a barplot of inclusion levels obtained from rMATS.

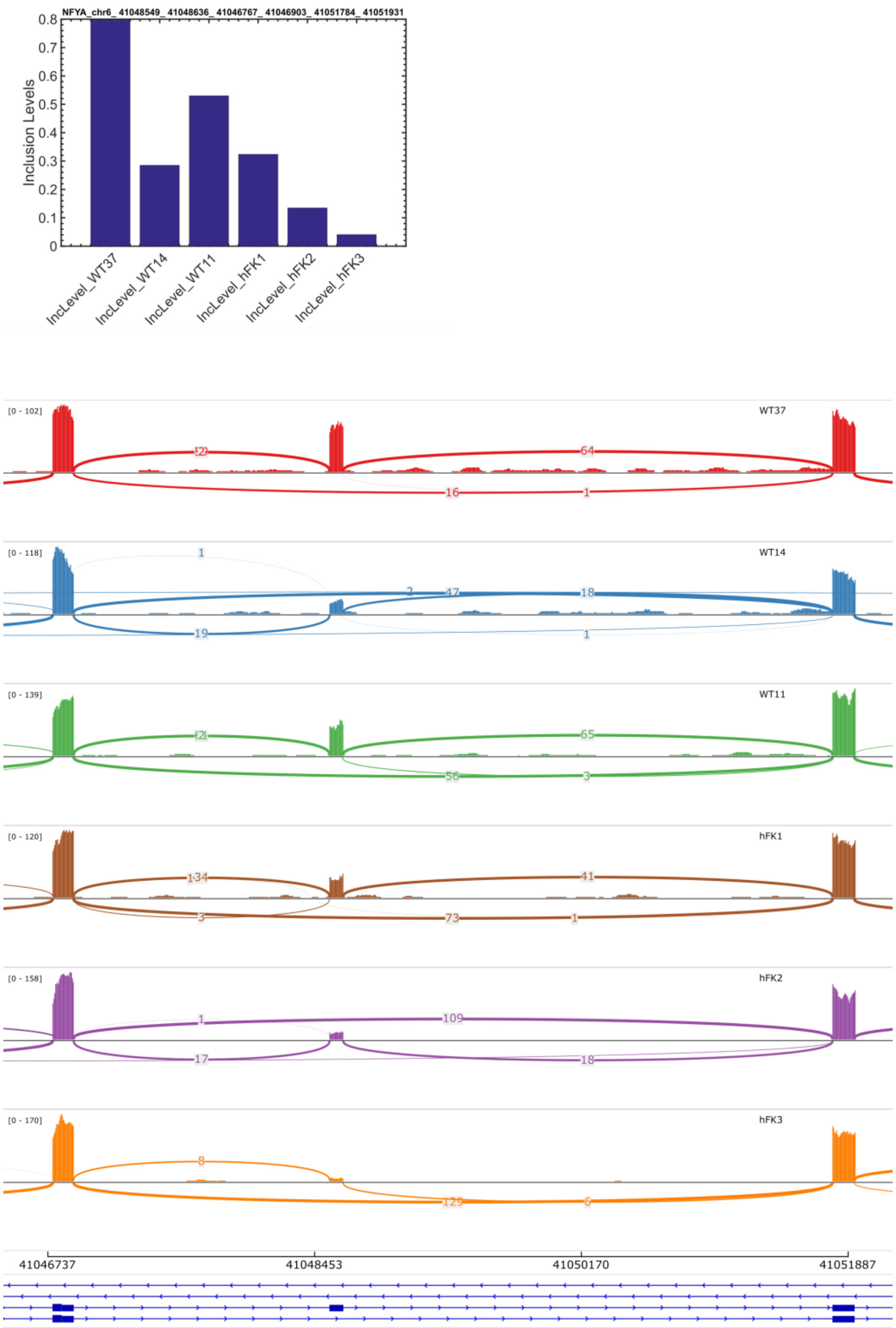

Figure S21: The gene NFYA contains a cassette exon (skipped exon, SE) that is highly expressed in the Wilms' tumor xenografts (WT37, WT14, and WT11) and the cells representing the early stage of human kidney development (hFK1), and whose expression gradually decreases during later stages of kidney development (hFK2 and hFK3). Shown are a Sashimi plot and a barplot of inclusion levels obtained from rMATS.

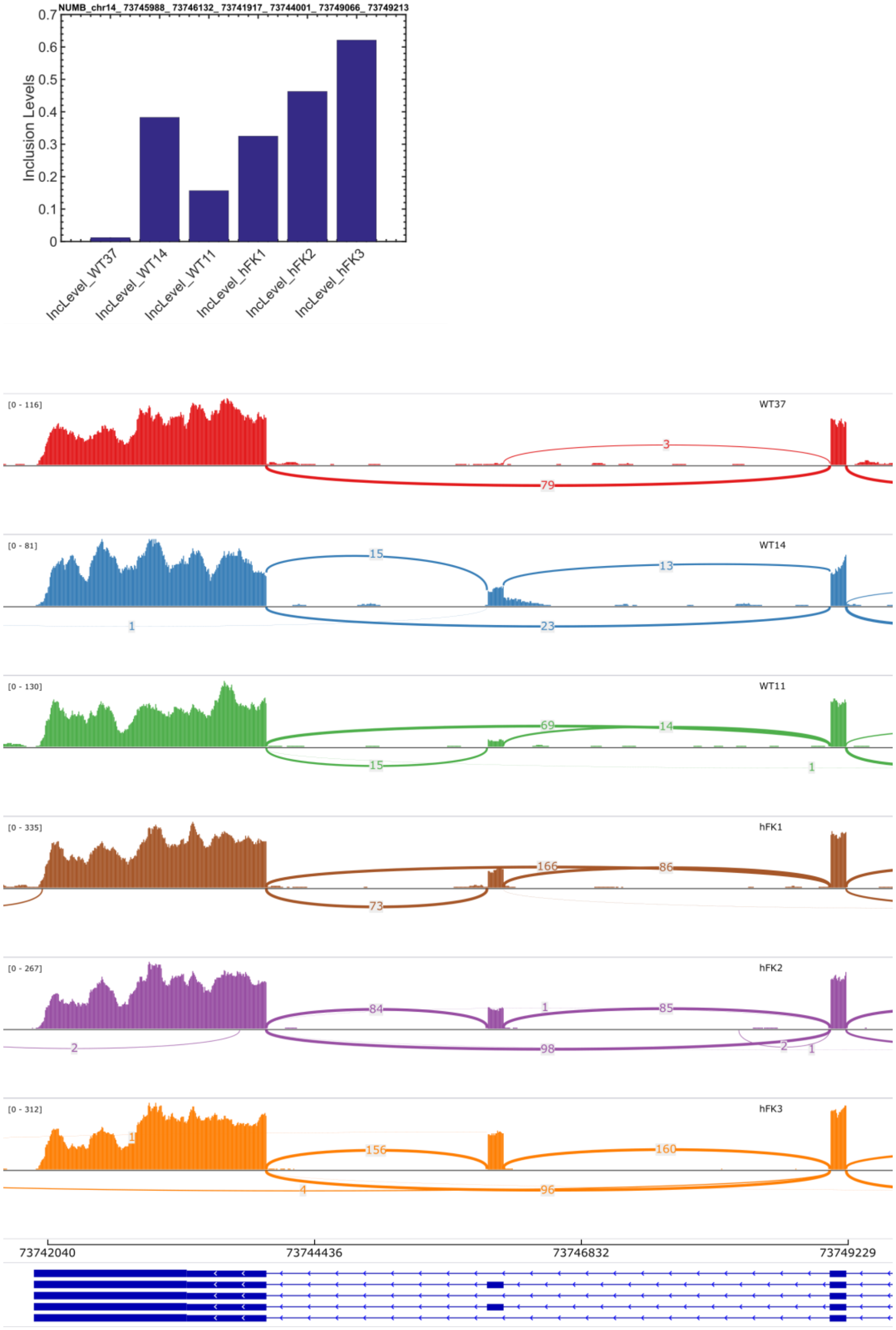

Figure S22: The gene NUMB contains a cassette exon (skipped exon, SE) that is lowly expressed in the Wilms' tumor xenografts (WT37, WT14, and WT11) and the cells representing the early stage of human kidney development (hFK1), and whose expression gradually increases during later stages of kidney development (hFK2 and hFK3). Shown are a Sashimi plot and a barplot of inclusion levels obtained from rMATS.

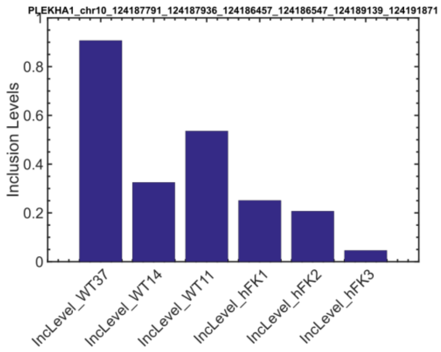

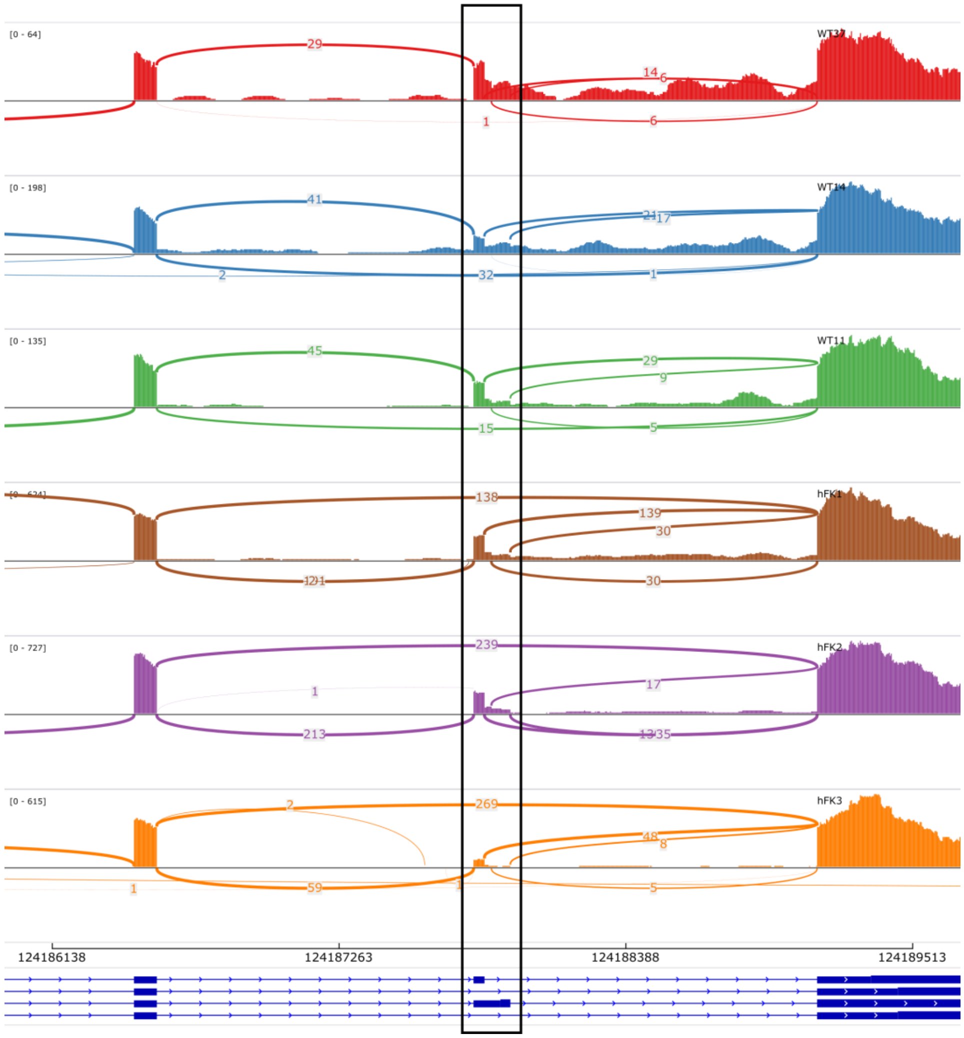

Figure S23: The gene PLEKHA1 contains a cassette exon (skipped exon, SE) that is highly expressed in the Wilms' tumor xenografts (WT37, WT14, and WT11) and the cells representing the early stage of human kidney development (hFK1), and whose expression gradually decreases during later stages of kidney development (hFK2 and hFK3). Shown are a Sashimi plot and a barplot of inclusion levels obtained from rMATS.

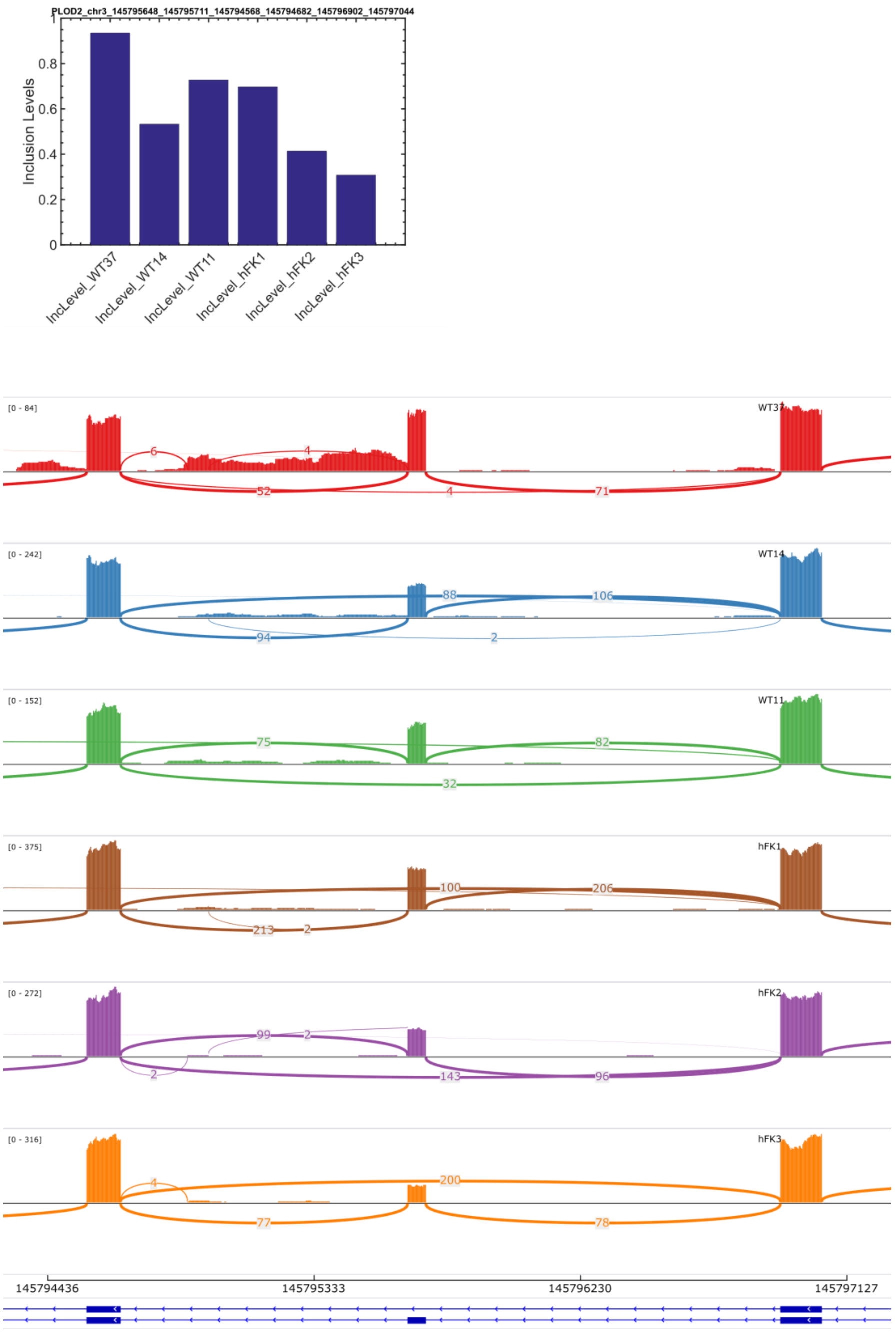

Figure S24: The gene PLOD2 [4,13,14], a gene for which alternative splicing was previously found to be regulated by the RNA binding protein RBFOX2 [4], contains a cassette exon (skipped exon, SE) that is highly expressed in the Wilms' tumor xenografts (WT37, WT14, and WT11) and the cells representing the early stage of human kidney development (hFK1), and whose expression gradually decreases during later stages of kidney development (hFK2 and hFK3). Shown are a Sashimi plot and a barplot of inclusion levels obtained from rMATS.

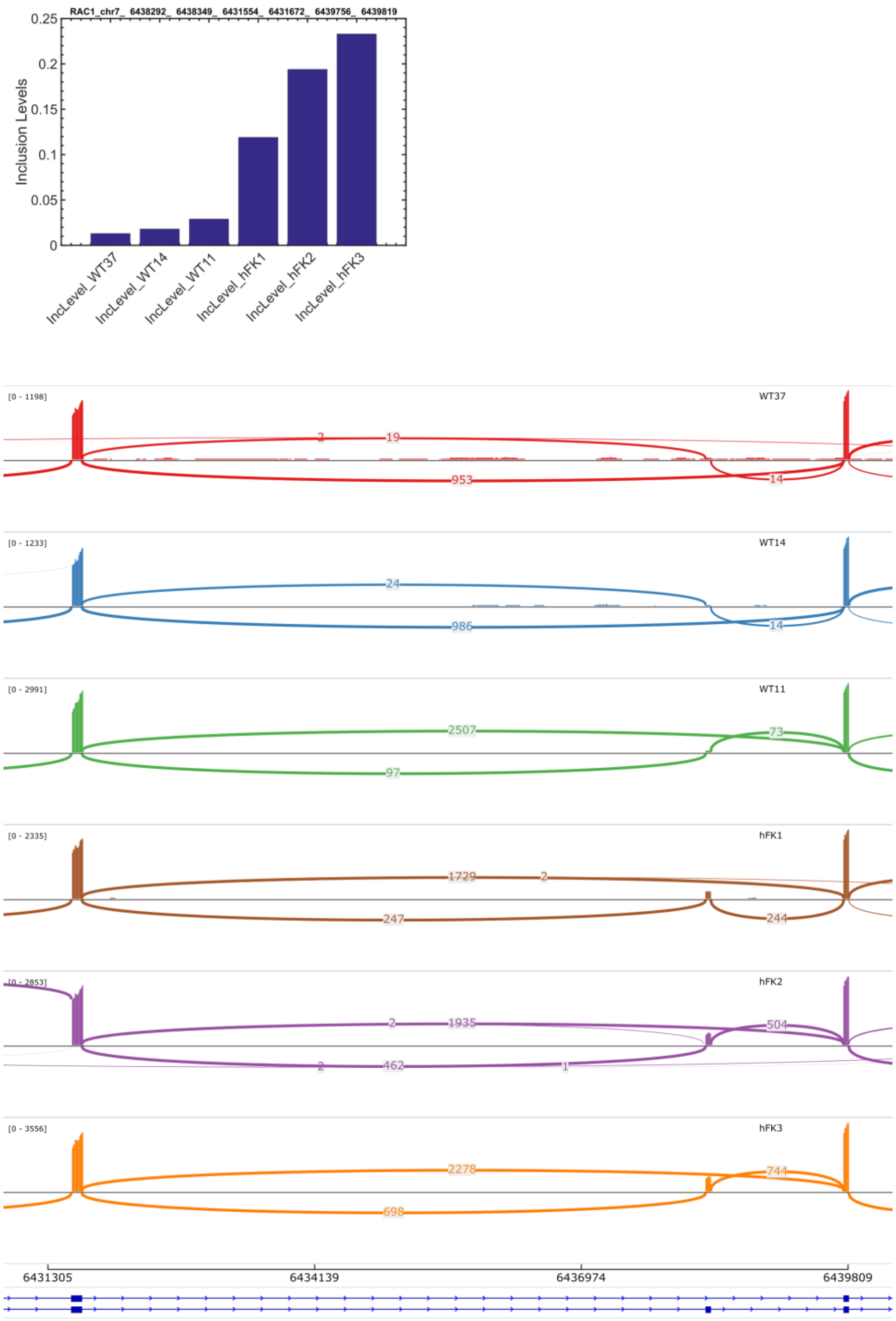

Figure S25: The gene RAC1 contains a cassette exon (skipped exon, SE) that is lowly expressed in the Wilms' tumor xenografts (WT37, WT14, and WT11) and the cells representing the early stage of human kidney development (hFK1), and whose expression gradually increases during later stages of kidney development (hFK2 and hFK3). Shown are a Sashimi plot and a barplot of inclusion levels obtained from rMATS.

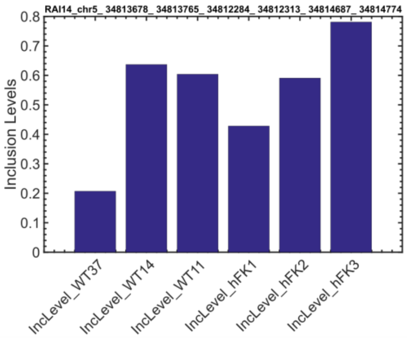

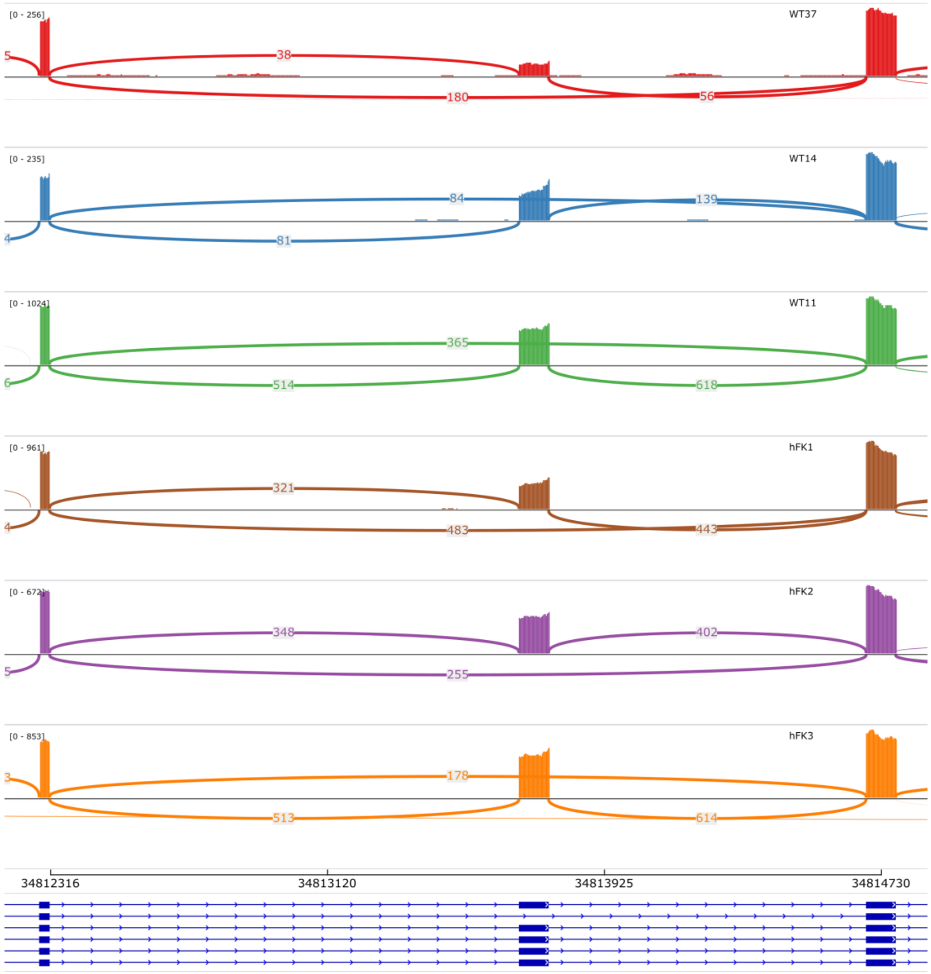

Figure S26: The gene RAI14 contains a cassette exon (skipped exon, SE) that is lowly or moderately expressed in the Wilms' tumor xenografts (WT37, WT14, and WT11) and the cells representing the early and intermediate stages of human kidney development (hFK1 and hFK2), and whose expression increases during the later stage of kidney development (hFK3). Shown are a Sashimi plot and a barplot of inclusion levels obtained from rMATS.

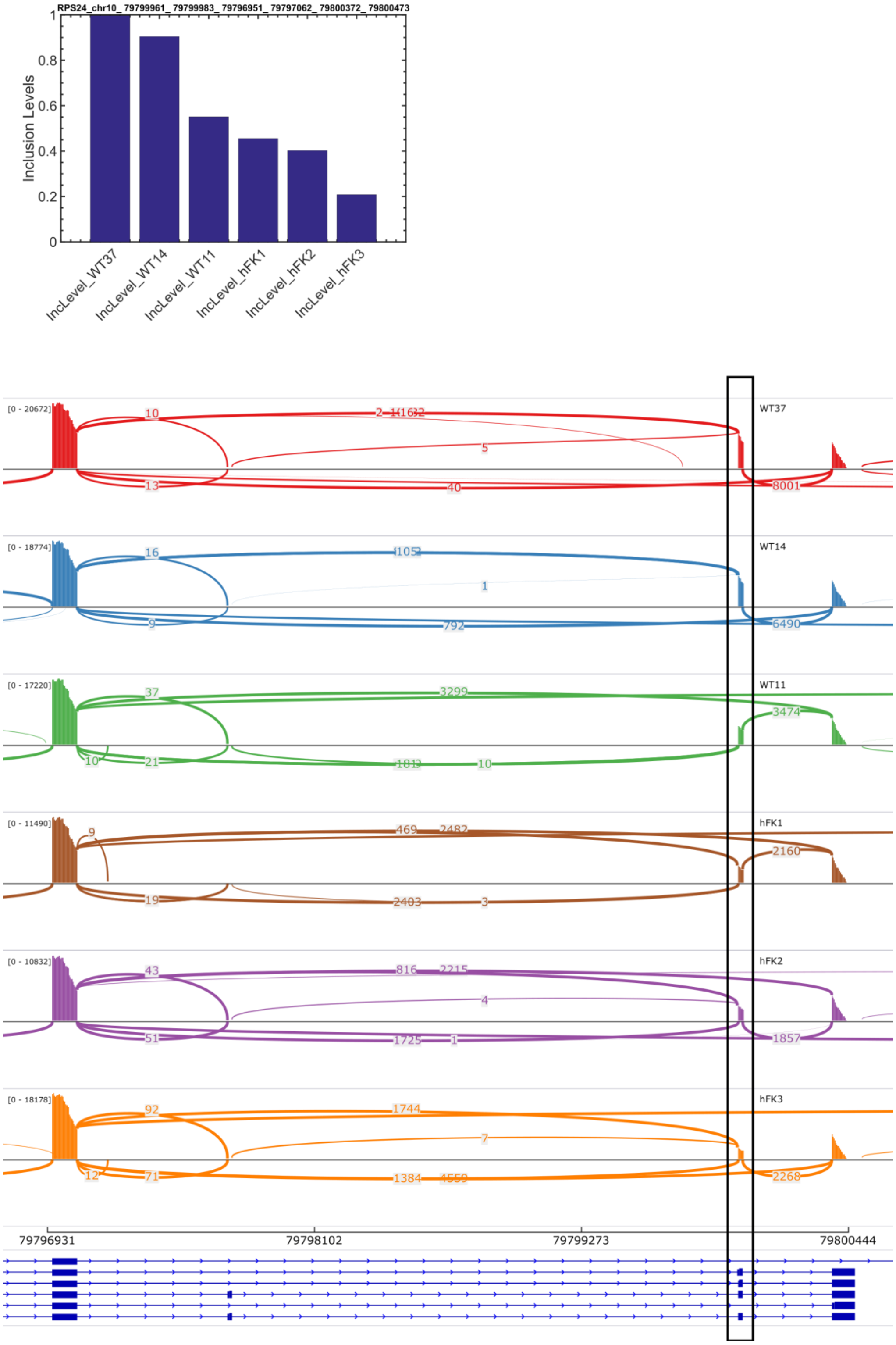

Figure S27: The gene RPS24 contains a cassette exon (skipped exon, SE) that is highly expressed in the Wilms' tumor xenografts (WT37, WT14, and WT11) and the cells representing the early stage of human kidney development (hFK1), and whose

expression gradually decreases during later stages of kidney development (hFK2 and hFK3). Shown are a Sashimi plot and a barplot of inclusion levels obtained from rMATS.

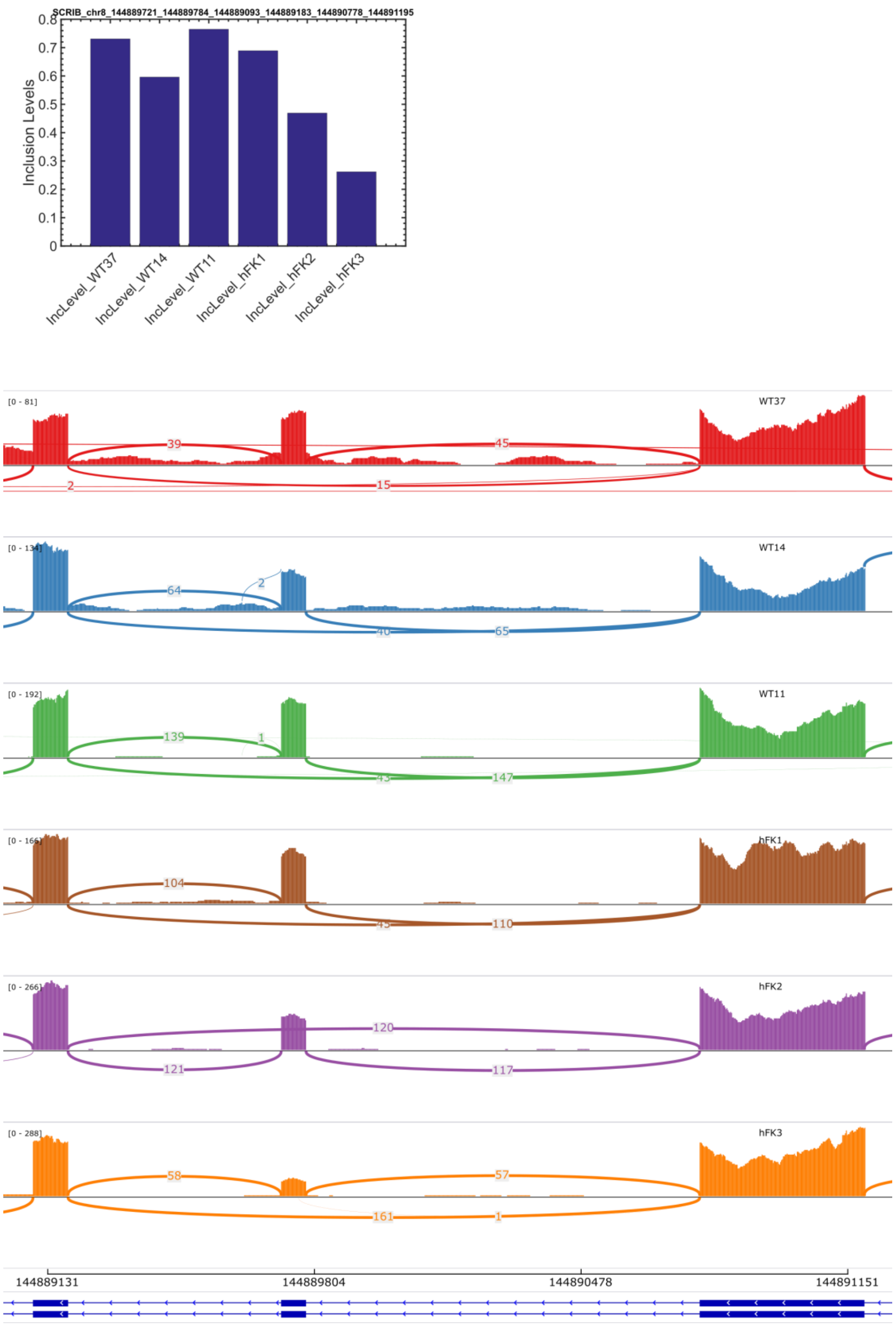

Figure S28: The gene SCRIB contains a cassette exon (skipped exon, SE) that is highly expressed in the Wilms' tumor xenografts (WT37, WT14, and WT11) and the cells representing the early stage of human kidney development (hFK1), and whose expression gradually decreases during later stages of kidney development (hFK2 and hFK3). Shown are a Sashimi plot and a barplot of inclusion levels obtained from rMATS.

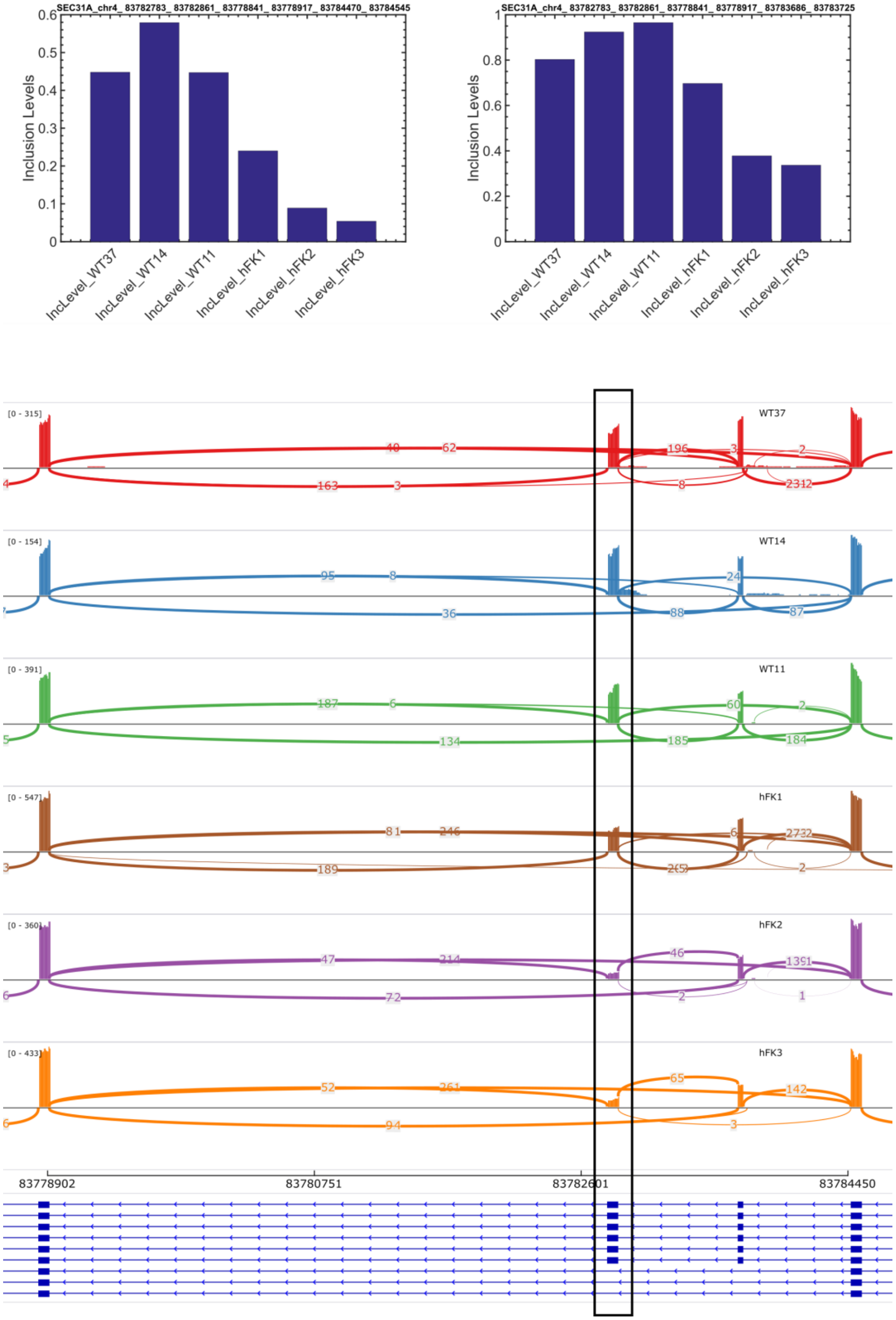

Figure S29: The gene SEC31A [18], that was previously shown to be alternatively spliced between mesenchymal and epithelial tissues, contains a cassette exon (skipped exon, SE) that is highly expressed in the Wilms' tumor xenografts (WT37, WT14, and WT11) and the cells representing the early stage of human kidney development (hFK1), and whose expression gradually decreases during later stages of kidney development (hFK2

and hFK3). Shown are a Sashimi plot and barplots of inclusion levels obtained from rMATS.

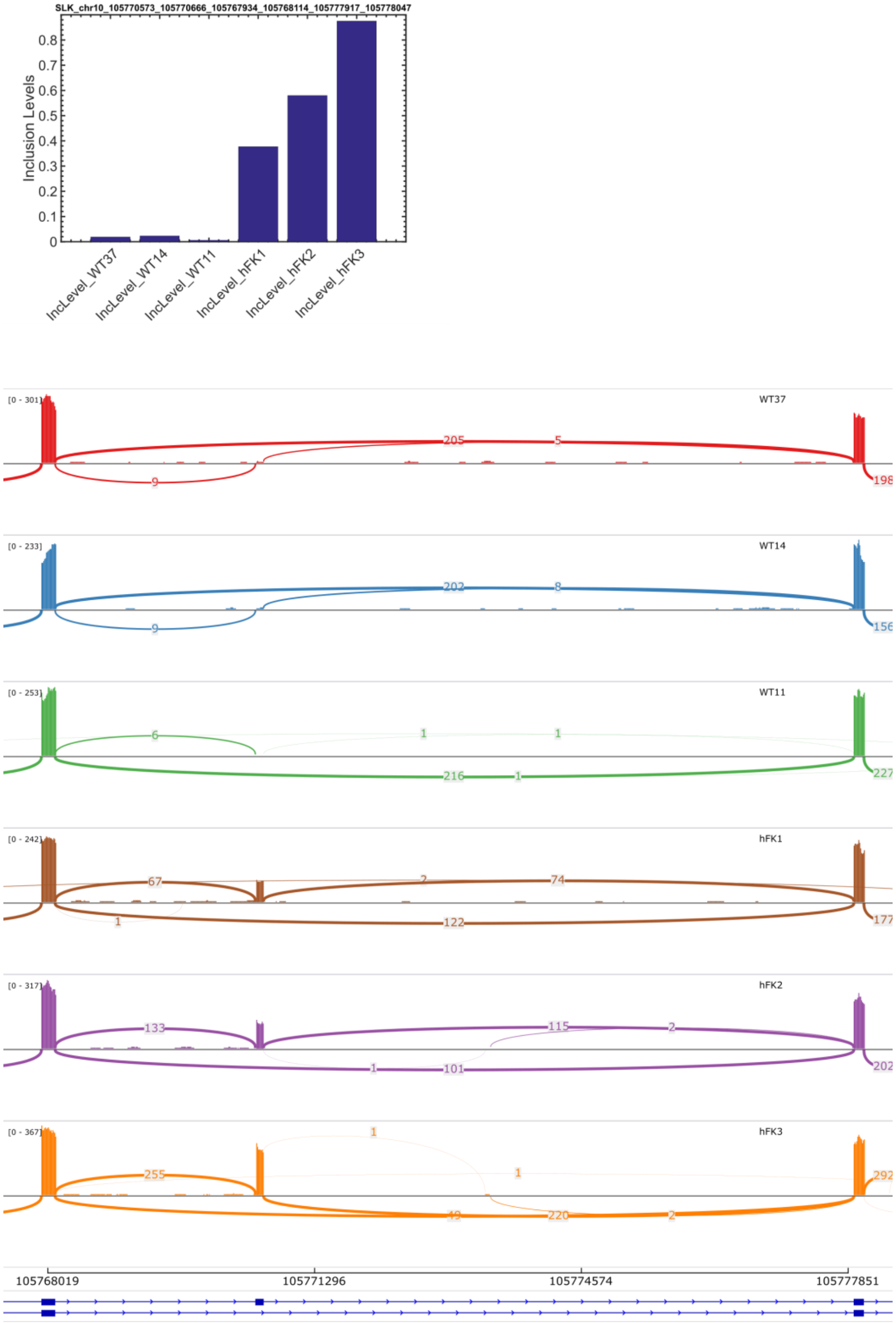

Figure S30: The gene SLK contains a cassette exon (skipped exon, SE) that is lowly expressed in the Wilms' tumor xenografts (WT37, WT14, and WT11) and the cells representing the early stage of human kidney development (hFK1), and whose expression gradually increases during later stages of kidney development (hFK2 and hFK3). Shown are a Sashimi plot and a barplot of inclusion levels obtained from rMATS.

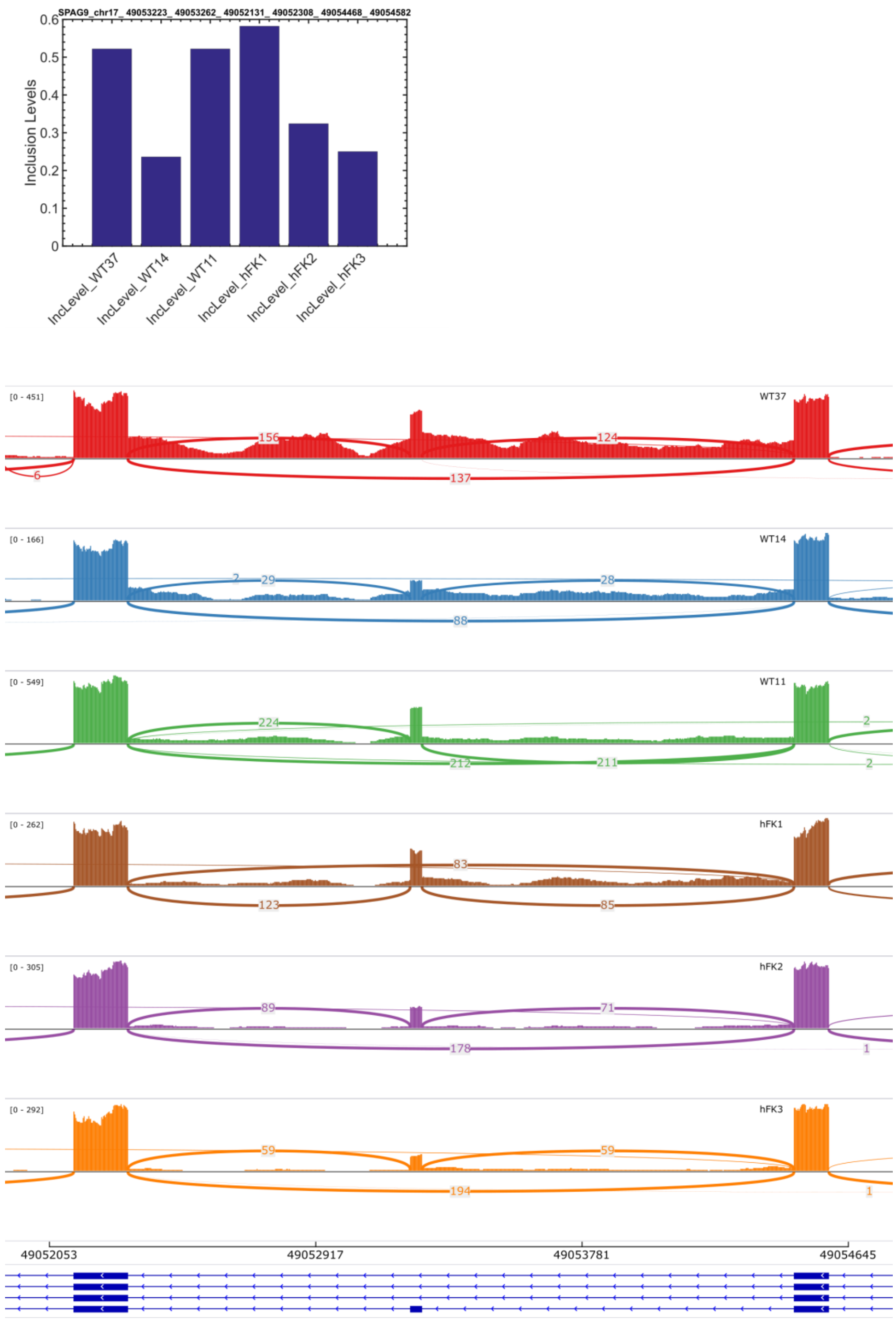

Figure S31: The gene SPAG9 contains a cassette exon (skipped exon, SE) that is highly expressed in two of the Wilms' tumor xenografts (WT37 and WT11) and the cells representing the early stage of human kidney development (hFK1), and whose expression gradually decreases during later stages of kidney development (hFK2 and hFK3). Shown are a Sashimi plot and a barplot of inclusion levels obtained from rMATS.

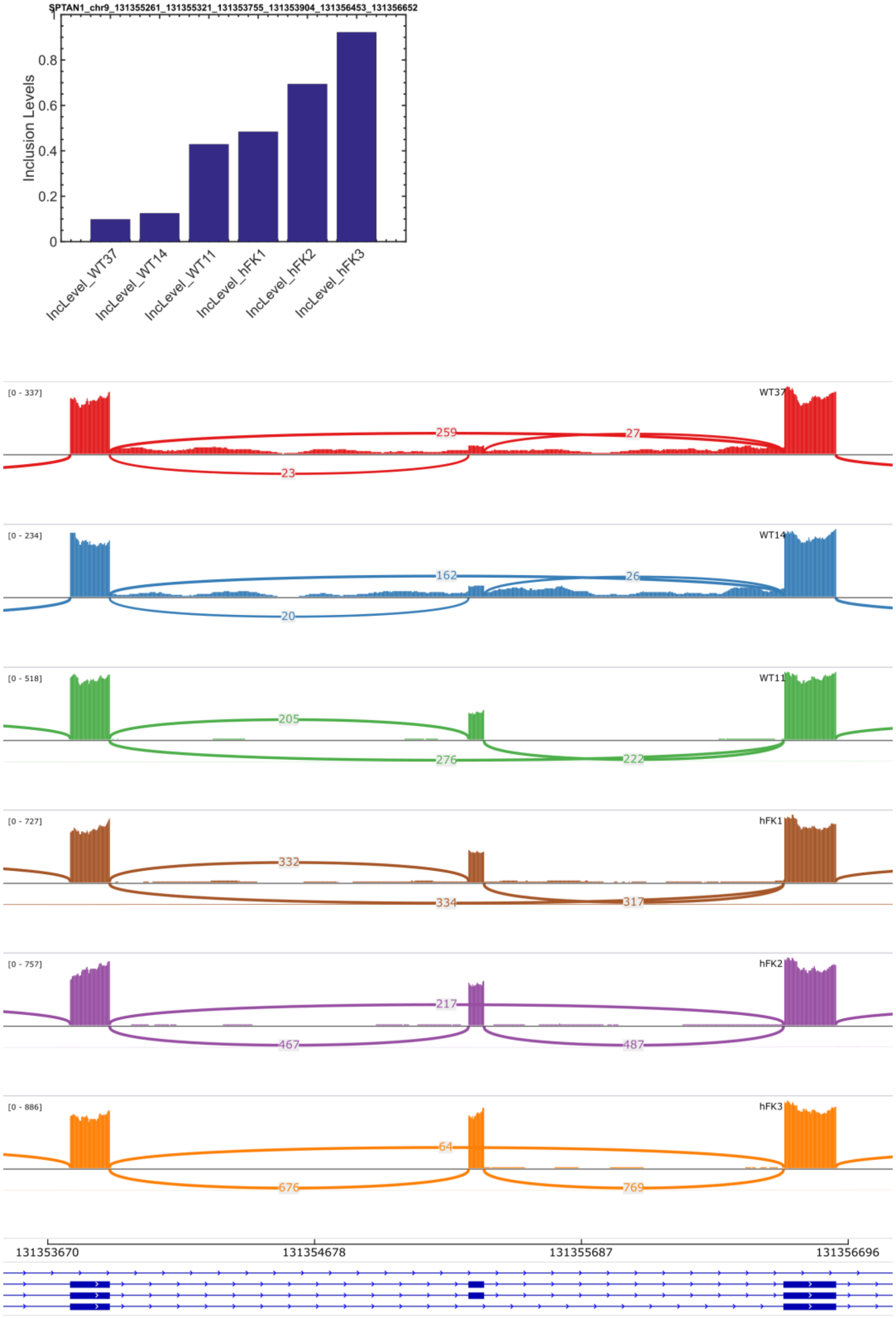

Figure S32: The gene SPTAN1 contains a cassette exon (skipped exon, SE) that is lowly expressed in the Wilms' tumor xenografts (WT37, WT14, and WT11) and the cells representing the early stage of human kidney development (hFK1), and whose expression gradually increases during later stages of kidney development (hFK2 and hFK3). Shown are a Sashimi plot and a barplot of inclusion levels obtained from rMATS.

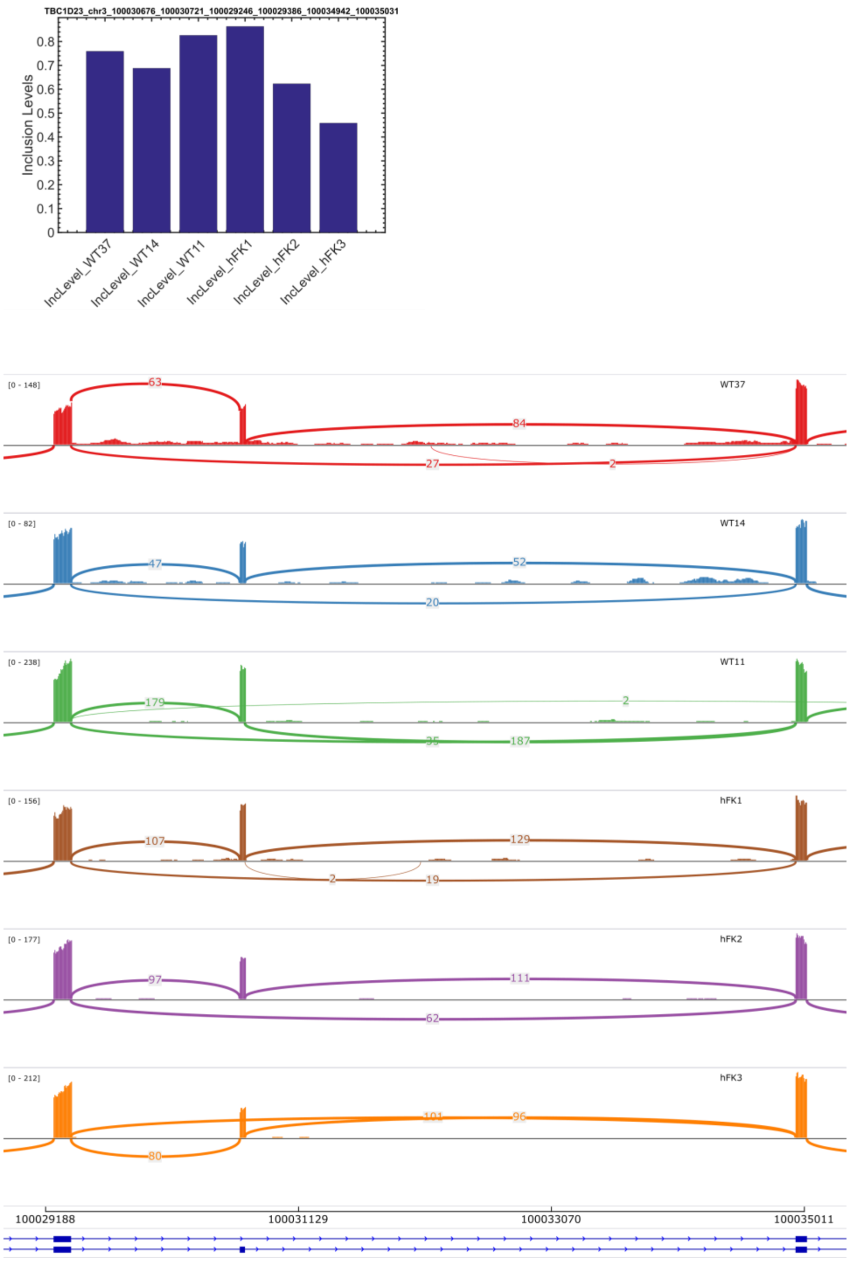

Figure S33: The gene TBC1D23 contains a cassette exon (skipped exon, SE) that is highly expressed in the Wilms' tumor xenografts (WT37, WT14, and WT11) and the cells representing the early stage of human kidney development (hFK1), and whose expression gradually decreases during later stages of kidney development (hFK2 and hFK3). Shown are a Sashimi plot and a barplot of inclusion levels obtained from rMATS.

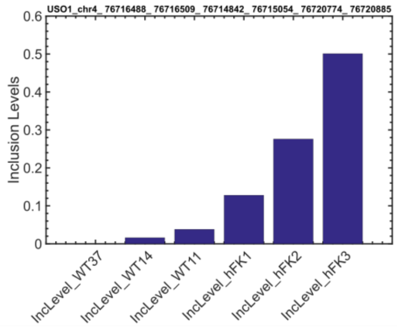

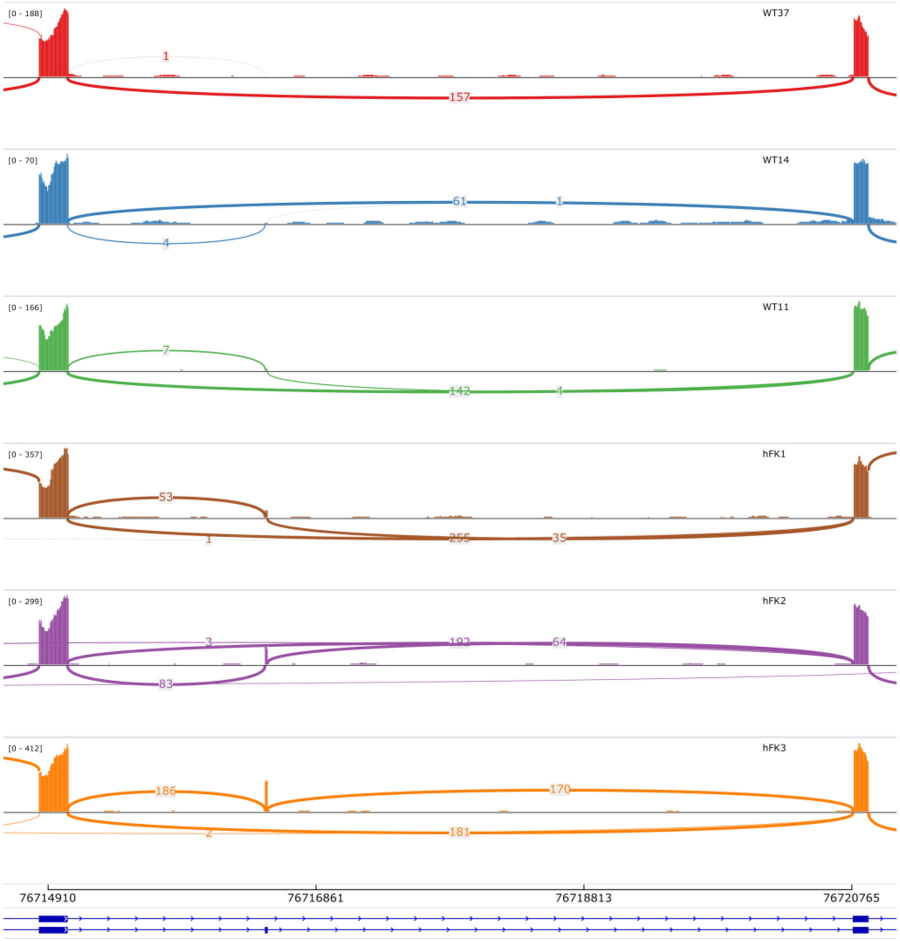

Figure S34: The gene USO1 contains a cassette exon (skipped exon, SE) that is lowly expressed in the Wilms' tumor xenografts (WT37, WT14, and WT11) and the cells representing the early stage of human kidney development (hFK1), and whose expression gradually increases during later stages of kidney development (hFK2 and hFK3). Shown are a Sashimi plot and a barplot of inclusion levels obtained from rMATS.

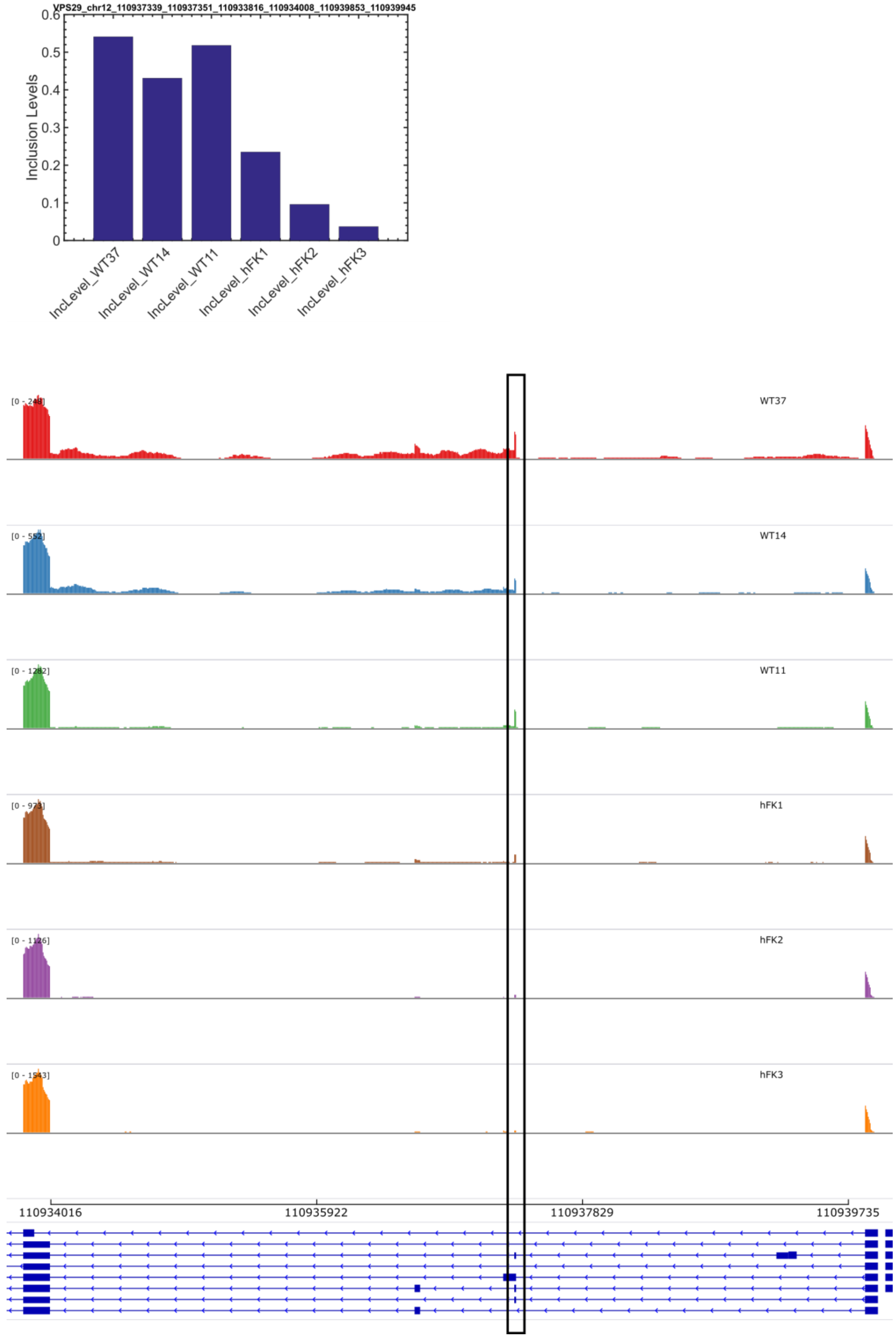

Figure S35: The gene VPS29 contains a cassette exon (skipped exon, SE) that is highly expressed in the Wilms' tumor xenografts (WT37, WT14, and WT11) and the cells representing the early stage of human kidney development (hFK1), and whose expression gradually decreases during later stages of kidney development (hFK2 and hFK3). Shown are a Sashimi plot and a barplot of inclusion levels obtained from rMATS.

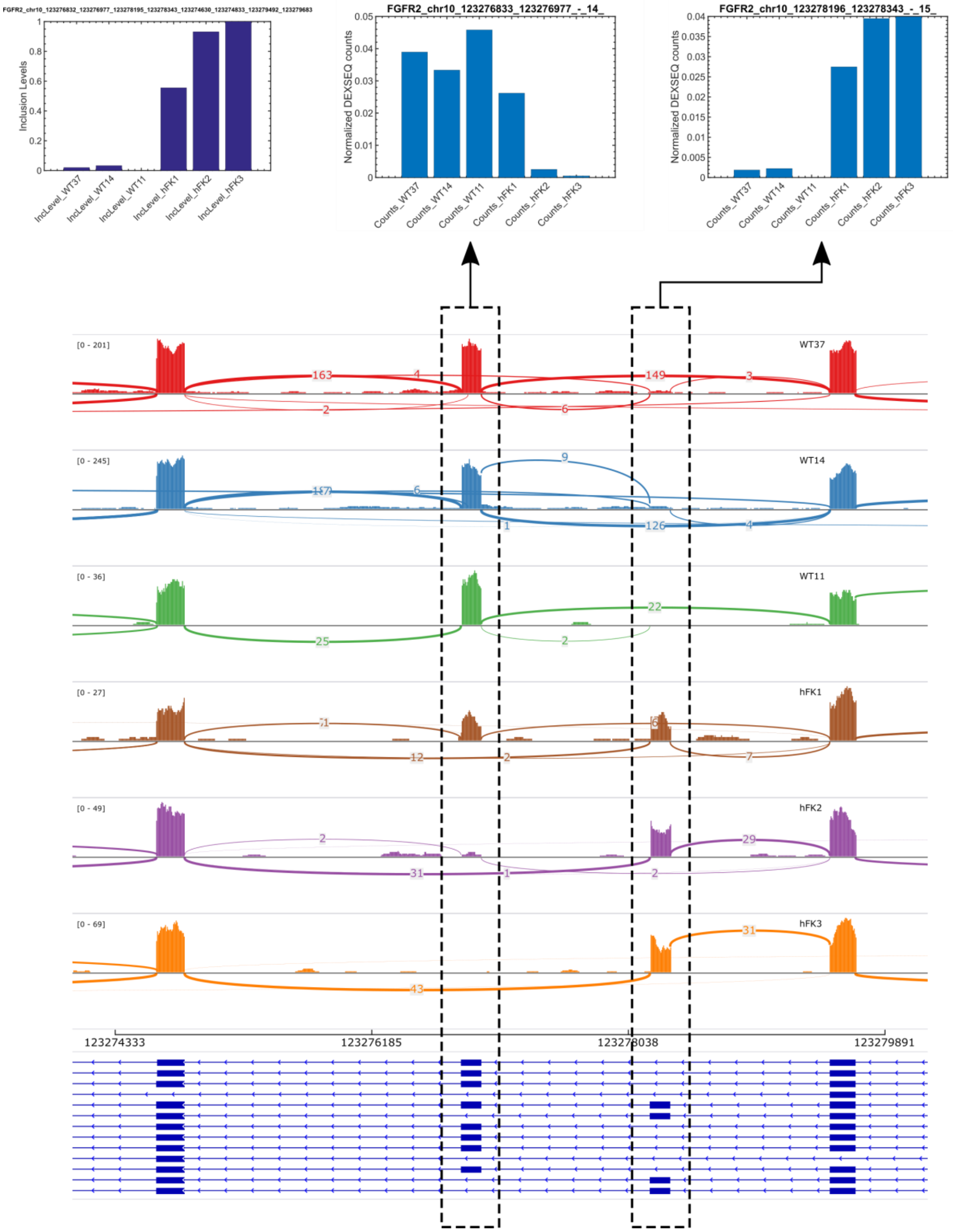

Figure S36: The gene FGFR2 [9,10], that was previously shown to be alternatively spliced between mesenchymal and epithelial tissues, contains two mutually exclusive exons (MXE), one of which is lowly expressed in the Wilms' tumor xenografts (WT37, WT14, and WT11) and the cells representing the early stage of human kidney development (hFK1), and whose expression gradually increases during later stages of kidney development (hFK2 and hFK3). Shown are a Sashimi plot, a barplot of inclusion levels obtained from rMATS, and barplots of normalized DEXseq counts.

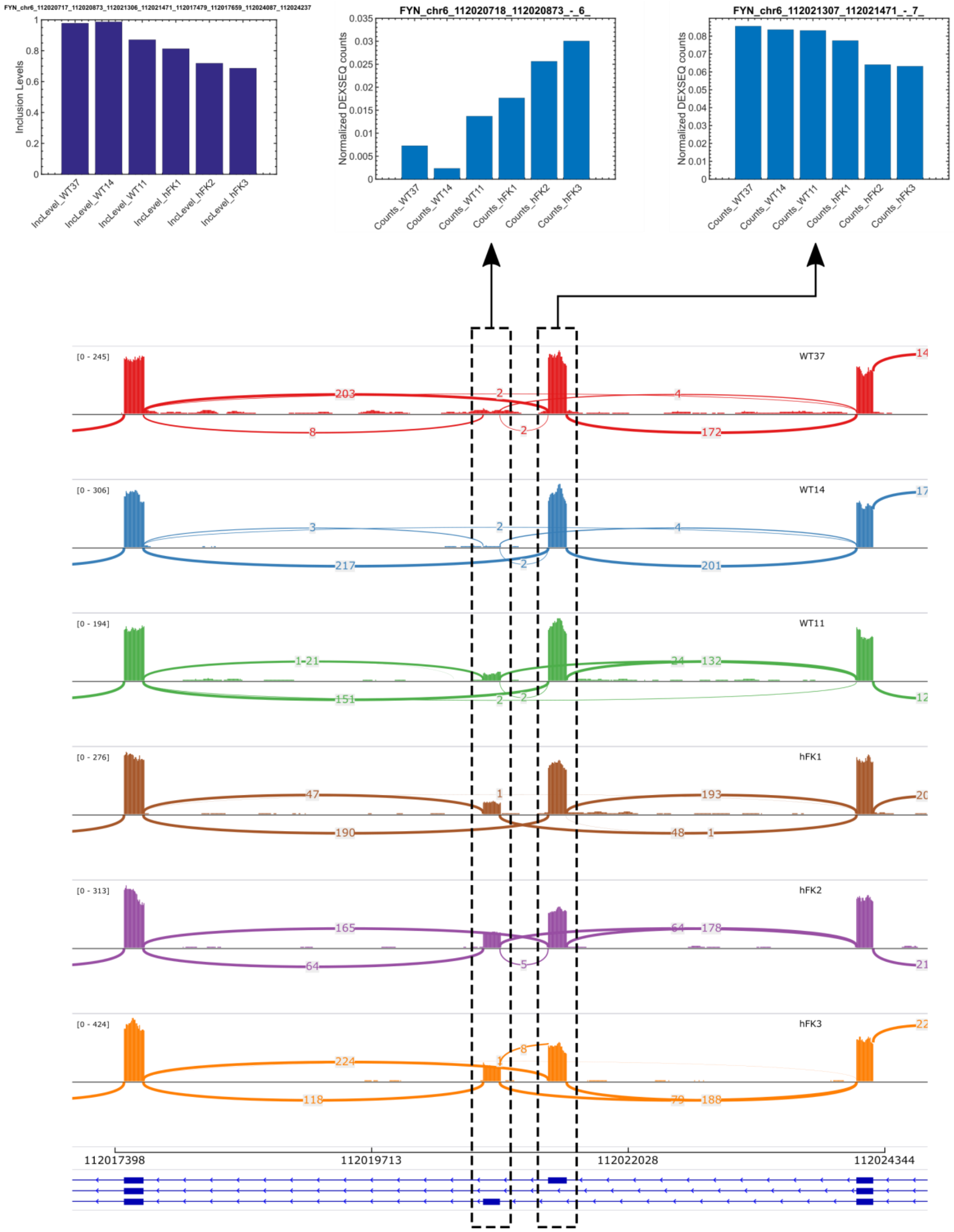

Figure S37: The gene FYN contains two mutually exclusive exons (MXE), one of which is lowly expressed in the Wilms' tumor xenografts (WT37, WT14, and WT11) and the cells representing the early stage of human kidney development (hFK1), and whose expression gradually increases during later stages of kidney development (hFK2 and hFK3). Shown are a Sashimi plot, a barplot of inclusion levels obtained from rMATS, and barplots of normalized DEXseq counts.

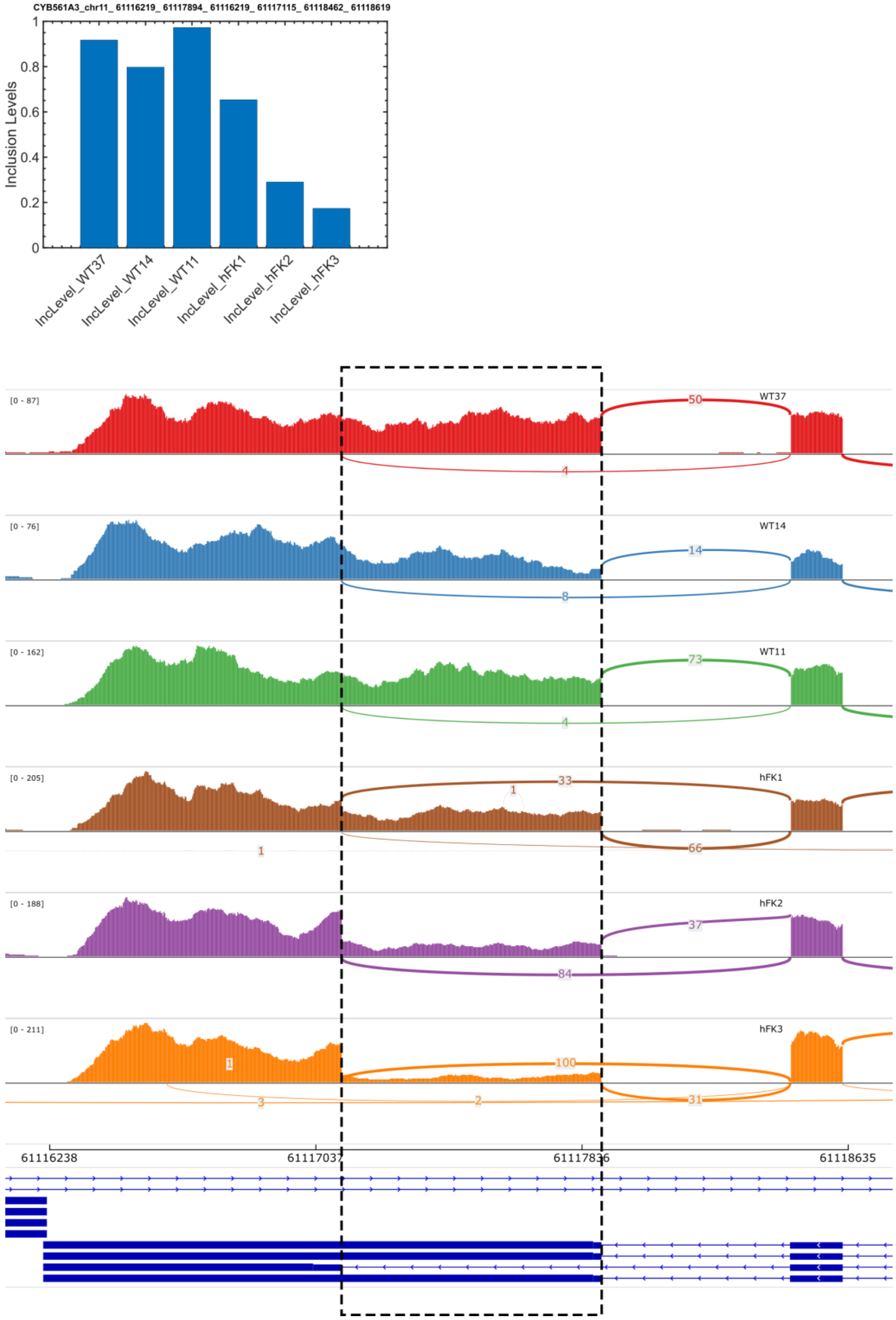

Figure S38: The gene CYB561A3 contains an alternative 3' splice-site (A3SS), with an exon segment that is highly expressed in the Wilms' tumor xenografts (WT37, WT14, and WT11) and the cells representing the early stage of human kidney development (hFK1), and whose expression gradually decreases during later stages of kidney development (hFK2 and hFK3). Shown are a Sashimi plot and a barplot of inclusion levels obtained from rMATS.

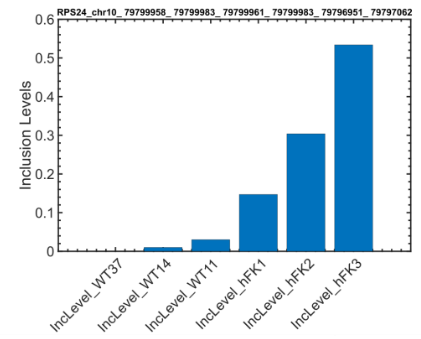

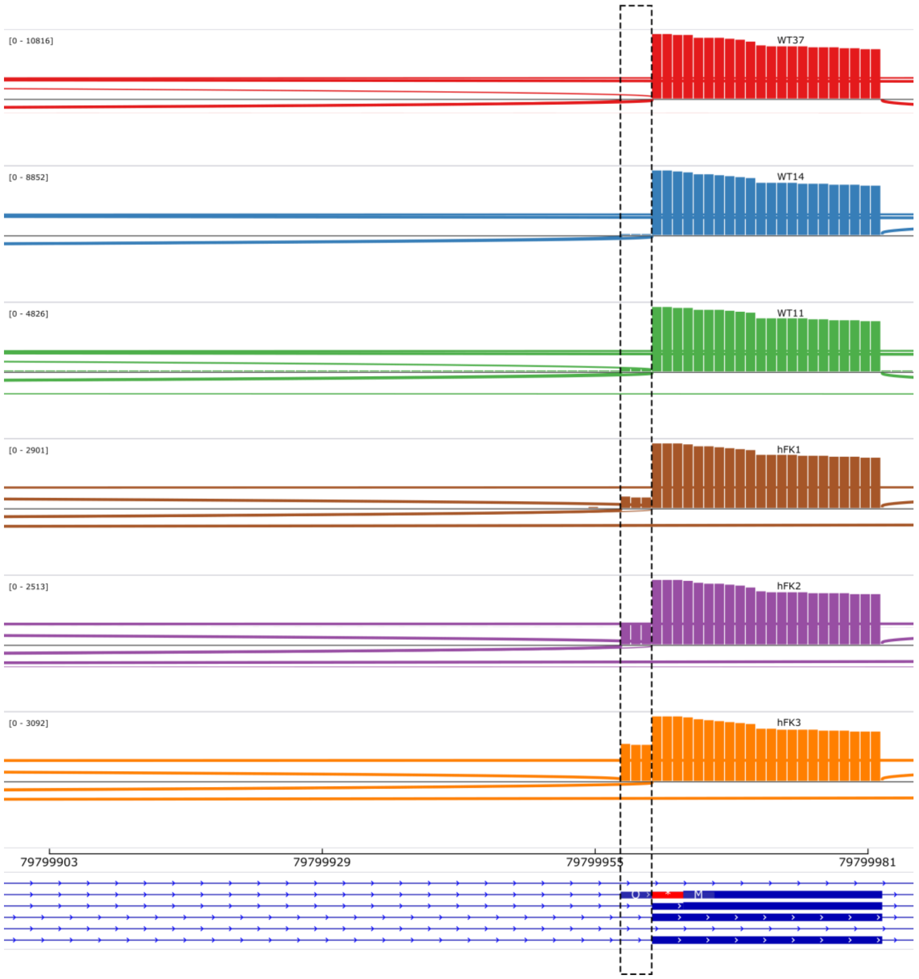

Figure S39: The gene RPS24 contains an alternative 3’ splice-site (A3SS), with an exon segment that is lowly expressed in the Wilms' tumor xenografts (WT37, WT14, and WT11) and the cells representing the early stage of human kidney development (hFK1), and whose expression gradually increases during later stages of kidney development (hFK2 and hFK3). Shown are a Sashimi plot and a barplot of inclusion levels obtained from rMATS.

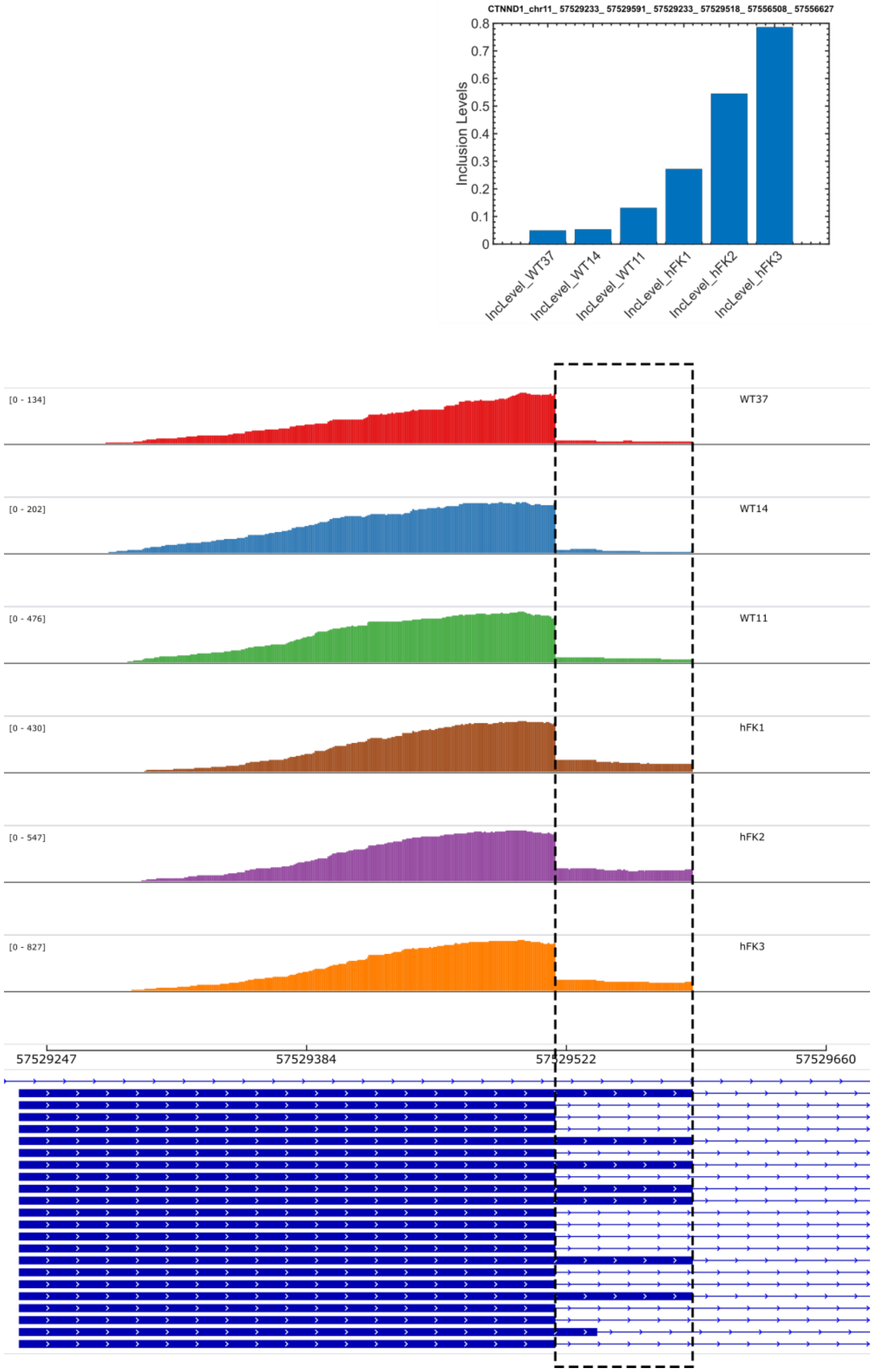

Figure S40: The gene CTNND1 contains an alternative 5' splice-site (A5SS), with an exon segment that is lowly expressed in the Wilms' tumor xenografts (WT37, WT14, and WT11) and the cells representing the early stage of human kidney development (hFK1), and whose expression gradually increases during later stages of kidney development (hFK2 and hFK3). Shown are a Sashimi plot and a barplot of inclusion levels obtained from rMATS.

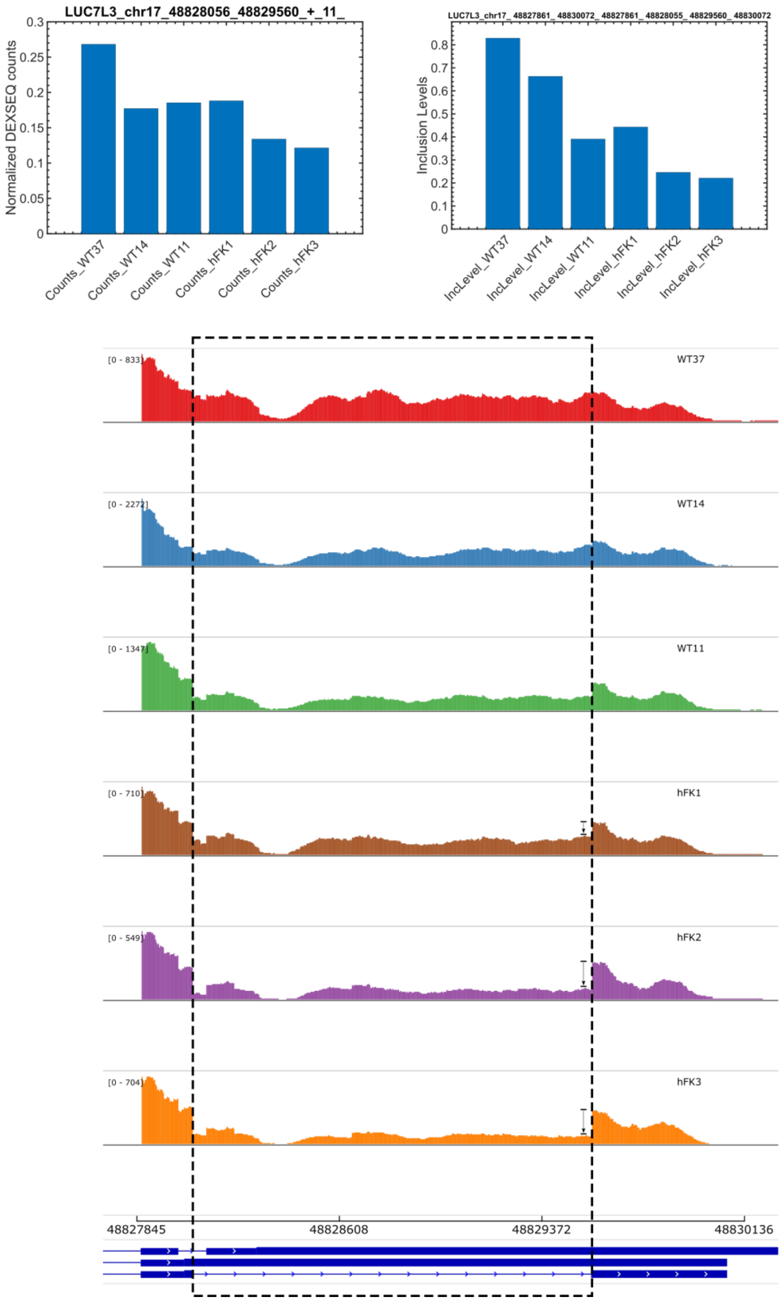

Figure S41: The gene LUC7L3 contains a retained intron (RI), with an exon segment that is highly expressed in the Wilms' tumor xenografts (WT37, WT14, and WT11) and the cells representing the early stage of human kidney development (hFK1), and whose expression gradually decreases during later stages of kidney development (hFK2 and hFK3). Shown are a Sashimi plot, a barplot of inclusion levels obtained from rMATS, and a barplot of normalized DEXseq counts.

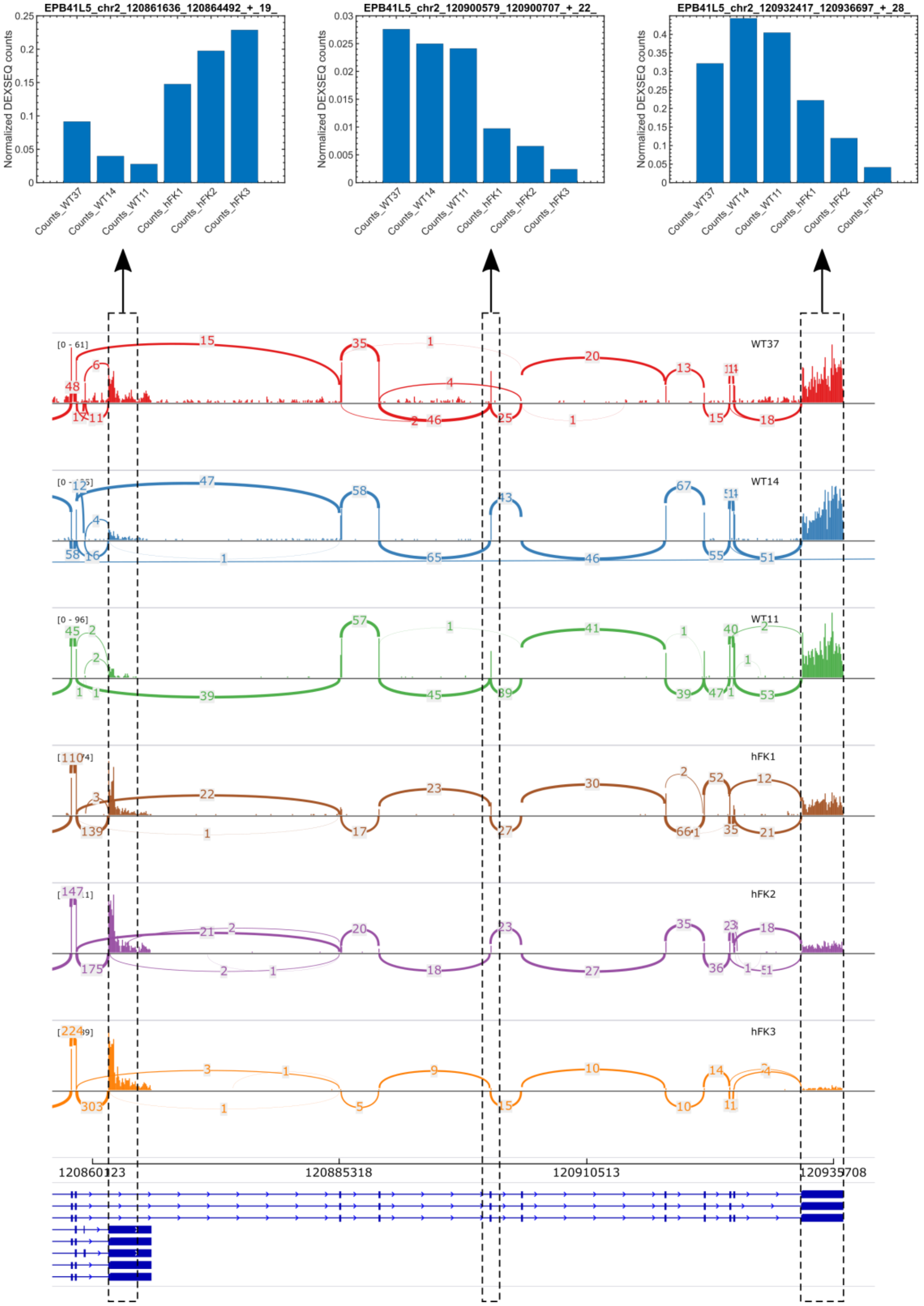

Figure S42: The gene EPB41L5 [6,11], that was previously shown to be alternatively spliced between mesenchymal and epithelial tissues, has two alternative 3' ends, with a long isoform that is highly expressed in the Wilms' tumor xenografts (WT37, WT14, and WT11) and the cells representing the early stage of human kidney development (hFK1), and whose expression gradually decreases during later stages of kidney development (hFK2 and hFK3). Shown are barplots of normalized DEXseq counts for selected exons from the long and short isoforms.

REFERENCES

1. Shen S, Park JW, Lu Z, Lin L, Henry MD, Wu YN, et al. rMATS: robust and flexible detection of differential alternative splicing from replicate RNA-Seq data. Proc Natl Acad Sci U S A. 2014;111: E5593-601. doi:10.1073/pnas.1419161111
2. Katz Y, Wang ET, Airoldi EM, Burge CB. Analysis and design of RNA sequencing experiments for identifying isoform regulation. Nat Methods. 2010;7: 1009–1015. doi:10.1038/nmeth.1528
3. Anders S, Reyes A, Huber W. Detecting differential usage of exons from RNA-seq data. Genome Res. 2012;22: 2008–2017. doi:10.1101/gr.133744.111.Freely
4. Shapiro IM, Cheng AW, Flytzanis NC, Balsamo M, Condeelis JS, Oktay MH, et al. An emt-driven alternative splicing program occurs in human breast cancer and modulates cellular phenotype. PLoS Genet. 2011;7. doi:10.1371/journal.pgen.1002218
5. Di Modugno F, Iapicca P, Boudreau A, Mottolese M, Terrenato I, Perracchio L, et al. Splicing program of human MENA produces a previously undescribed isoform associated with invasive, mesenchymal-like breast tumors. Proc Natl Acad Sci U S A. 2012;109: 19280–5. doi:10.1073/pnas.1214394109
6. Warzecha CC, Shen S, Xing Y, Carstens RP. The epithelial splicing factors ESRP1 and ESRP2 positively and negatively regulate diverse types of alternative splicing events. RNA Biol. 2009;6: 546–562. doi:10.4161/rna.6.5.9606
7. Sneath RJ, Mangham DC. The normal structure and function of CD44 and its role in neoplasia. Mol Pathol. 1998;51: 191–200. doi:10.1136/mp.51.4.191
8. Brown RL, Reinke LM, Damerow MS, Perez D, Chodosh L a., Yang J, et al. CD44 splice isoform switching in human and mouse epithelium is essential for epithelial-mesenchymal transition and breast cancer progression. J Clin Invest. 2011;121: 1064–1074. doi:10.1172/JCI44540
9. Warzecha CC, Sato TK, Nabet B, Hogenesch JB, Carstens RP. ESRP1 and ESRP2 Are Epithelial Cell-Type-Specific Regulators of FGFR2 Splicing. Mol Cell. Elsevier Ltd; 2009;33: 591–601. doi:10.1016/j.molcel.2009.01.025
10. Hovhannisyan RH, Warzecha CC, Carstens RP. Characterization of sequences and mechanisms through which ISE/ISS-3 regulates FGFR2 splicing. Nucleic Acids Res. 2006;34: 373–385. doi:10.1093/nar/gkj407
11. Warzecha CC, Carstens RP. Complex changes in alternative pre-mRNA splicing play a central role in the epithelial-to-mesenchymal transition (EMT). Semin Cancer Biol. Elsevier Ltd; 2012;22: 417–427. doi:10.1016/j.semcancer.2012.04.003
12. Braeutigam C, Rago L, Rolke A, Waldmeier L, Christofori G, Winter J. The RNA-binding protein Rbfox2: An essential regulator of EMT-driven alternative splicing and a mediator of cellular invasion. Oncogene. Nature Publishing Group; 2014;33: 1082–1092. doi:10.1038/onc.2013.50
13. Yeowell HN, Walker LC. Tissue specificity of a new splice form of the human lysyl hydroxylase 2 gene. Matrix Biol. 1999;18: 179–187. doi:10.1016/S0945-053X(99)00013-X

14. Yang Y, Park JW, Bebee TW, Warzecha CC, Guo Y, Shang X, et al. Determination of a Comprehensive Alternative Splicing Regulatory Network and Combinatorial Regulation by Key Factors during the Epithelial-to-Mesenchymal Transition. Mol Cell Biol. 2016;36: 1704–1719. doi:10.1128/MCB.00019-16
15. Bebee TW, Park JW, Sheridan KI, Warzecha CC, Cieply BW, Rohacek AM, et al. The splicing regulators Esrp1 and Esrp2 direct an epithelial splicing program essential for mammalian development. Elife. 2015;4: 1–27. doi:10.7554/eLife.08954
16. Bangru S, Arif W, Seimetz J, Bhate A, Chen J, Rashan EH, et al. Alternative splicing rewires Hippo signaling pathway in hepatocytes to promote liver regeneration. Nat Struct Mol Biol. 2018;25: 928–939. doi:10.1038/s41594-018-0129-2
17. Bhate A, Parker DJ, Bebee TW, Ahn J, Arif W, Rashan EH, et al. ESRP2 controls an adult splicing programme in hepatocytes to support postnatal liver maturation. Nat Commun. Nature Publishing Group; 2015;6: 8768. doi:10.1038/ncomms9768
18. Xu Y, Gao XD, Lee J-H, Huang H, Tan H, Ahn J, et al. Cell type-restricted activity of hnRNPM promotes breast cancer metastasis via regulating alternative splicing. Genes Dev. 2014;28: 1191–203. doi:10.1101/gad.241968.114
